# Nanotherapy delivery of c-myc inhibitor targets Protumor Macrophages and preserves Antitumor Macrophages in Breast Cancer

**DOI:** 10.7150/thno.44523

**Published:** 2020-06-12

**Authors:** Alison K. Esser, Michael H. Ross, Francesca Fontana, Xinming Su, Ariel Gabay, Gregory C. Fox, Yalin Xu, Jingyu Xiang, Anne H. Schmieder, Xiaoxia Yang, Grace Cui, Michael Scott, Samuel Achilefu, Jay Chauhan, Steven Fletcher, Gregory M. Lanza, Katherine N. Weilbaecher

**Affiliations:** 1 Department of Medicine, Division of Molecular Oncology, Washington University School of Medicine, St. Louis, MO 63110, USA.; 2 Department of Medicine, Division of Cardiology, Washington University School of Medicine, St. Louis, MO, 63110, USA.; 3 Department of Radiology, Washington University School of Medicine, St. Louis, MO, 63110, USA.; 4 Department of Pharmaceutical Sciences, University of Maryland School of Pharmacy, Baltimore, MD, 21201, USA.

**Keywords:** MYC, β3 integrin, nanoparticles, breast cancer, macrophages, drug delivery

## Abstract

Tumor-associated macrophages (TAMs) enhance tumor growth in mice and are correlated with a worse prognosis for breast cancer patients. While early therapies sought to deplete all macrophages, current therapeutics aim to reprogram pro-tumor macrophages (M2) and preserve those necessary for anti-tumor immune responses (M1). Recent studies have shown that c-MYC (MYC) is induced in M2 macrophages *in vitro* and *in vivo* where it regulates the expression of tumor-promoting genes. In a myeloid lineage MYC KO mouse model, MYC had important roles in macrophage maturation and function leading to reduced tumor growth. We therefore hypothesized that targeted delivery of a MYC inhibitor to established M2 TAMs could reduce polarization toward an M2 phenotype in breast cancer models.

**Methods:** In this study, we developed a MYC inhibitor prodrug (MI3-PD) for encapsulation within perfluorocarbon nanoparticles, which can deliver drugs directly to the cytosol of the target cell through a phagocytosis independent mechanism. We have previously shown that M2-like TAMs express significant levels of the vitronectin receptor, integrin β3, and *in vivo* targeting and therapeutic potential was evaluated using αvβ3 integrin targeted rhodamine-labeled nanoparticles (NP) or integrin αvβ3-MI3-PD nanoparticles.

**Results:** We observed that rhodamine, delivered by αvβ3-rhodamine NP, was incorporated into M2 tumor promoting macrophages through both phagocytosis-independent and dependent mechanisms, while NP uptake in tumor suppressing M1 macrophages was almost exclusively through phagocytosis. In a mouse model of breast cancer (4T1-GFP-FL), M2-like TAMs were significantly reduced with αvβ3-MI3-PD NP treatment. To validate this effect was independent of drug delivery to tumor cells and was specific to the MYC inhibitor, mice with integrin β3 knock out tumors (PyMT-Bo1 β3KO) were treated with αvβ3-NP or αvβ3-MI3-PD NP. M2 macrophages were significantly reduced with αvβ3-MI3-PD nanoparticle therapy but not αvβ3-NP treatment.

**Conclusion:** These data suggest αvβ3-NP-mediated drug delivery of a c-MYC inhibitor can reduce protumor M2-like macrophages while preserving antitumor M1-like macrophages in breast cancer.

## Introduction

New breast cancer therapies have significantly improved patient outcomes over the last decade, but for some subtypes or advanced malignancies, there are limited therapeutic options. Immunotherapies have primarily focused on enhanced T cell activation and increased cytotoxicity towards cancer cells. However, many breast cancers have high numbers of tumor-associated macrophages (TAMs) which, in addition to promoting tumor growth, repress antitumor T cell responses, correlate with poor prognosis, and limit the efficacy of immunotherapy 1, 2. Previous work has shown that macrophage depletion reduces tumor size in breast and other cancers 3.

Tumor-associated macrophages encompass a spectrum of subtypes with diverse functions, but are commonly divided into two broad categories: classically polarized M1 phenotype (antitumor) macrophages and alternatively polarized M2 phenotype (protumor) macrophages. Inherently cleared by the macrophage monocyte phagocytic system (MPS), nanoparticle technologies have been used as macrophage imaging agents (SPIO NP) 4, 5 and therapeutics 6-9. Iron oxide nanoparticles have been a dominant component in many of these nanosystems due to their ability to influence macrophages toward an M1 phenotype, increase M1/M2 ratios, and promote anti-tumor immune responses 10, 11. Examples of iron oxide containing nanoparticles that repolarize macrophages include *ex vivo* hyaluronic acid decorated SPIO NP (HIONs) 12 and immune stimulatory formulations; TLR3 agonist poly (I:C) 13, melanin-like iron oxide NP (Fe@PDA-PEG) 14, photogeneration of reactive oxygen species 15 and iron oxide nanoparticles under AMF exposure 16-18. Encapsulation of therapeutic cargo that inhibits proteins or genes specific to M2 macrophages or TAM-suppressive functions can further improve specificity 19.

In myeloid cells, the b-HLHZIP transcription factor c-MYC (MYC) has been shown to regulate macrophage inflammatory responses, macrophage maturation and M2 polarization, and tumor-promoting functions 20, 21. Therapeutic targeting of MYC in TAMs could therefore reduce the ability of macrophages to polarize to an immune suppressive M2 phenotype and enhance the switch to an inflammatory response. Previous attempts at inhibiting MYC function have included anti-sense nucleic acid strategies 22, RNA interference 23, and interference with MYC-MAX dimerization and subsequent E-box binding using small molecules 24-32. Several small-molecule inhibitors of the MYC-MAX interaction have been reported 24, 33-36 but all were challenged by rapid metabolism and poor bioavailability, leading to poor anti-tumor responses. To overcome these barriers, we used a potent small molecule inhibitor that we designed into a lipase-labile phosphatidylcholine prodrug, which enables stable incorporation into the phospholipid membrane of targeted perfluorocarbon nanoparticles 37 (See Supplemental Data).

For the present experimental work, we designed a MYC inhibitor prodrug (MI3-PD) for perfluorocarbon nanoparticle delivery to M2 macrophages through activated integrin αvβ3 with the intent to disrupt M2 polarization without compromising macrophage viability. We found that human breast cancer patient tumors have increased numbers of integrin αvβ3-positive macrophages, and we provide new evidence that human breast cancer TAMs express MYC. We also show that αvβ3-targeted nanoparticles at least in part, are taken up by a phagocytosis-independent mechanism in M2 macrophages. In murine immunocompetent models of estrogen receptor positive (ER+) and triple-negative breast cancers, αvβ3-targeted MI3 prodrug nanoparticles (αvβ3-MI3-PD NP) decreased M2 polarized TAMs in mammary fat pad tumors and preserved M1 TAM numbers. These data provide therapeutic proof of principle that inhibition of MYC signaling through αvβ3-targeted drug delivery of the small molecule MI3-PD could be used to reduce M2 macrophages in the tumor microenvironment while sparing M1 antitumor macrophages.

## Materials and Methods

### Synthesis and characterization of αvβ3-targeted-MI3-PD NP

**Figure 1:**
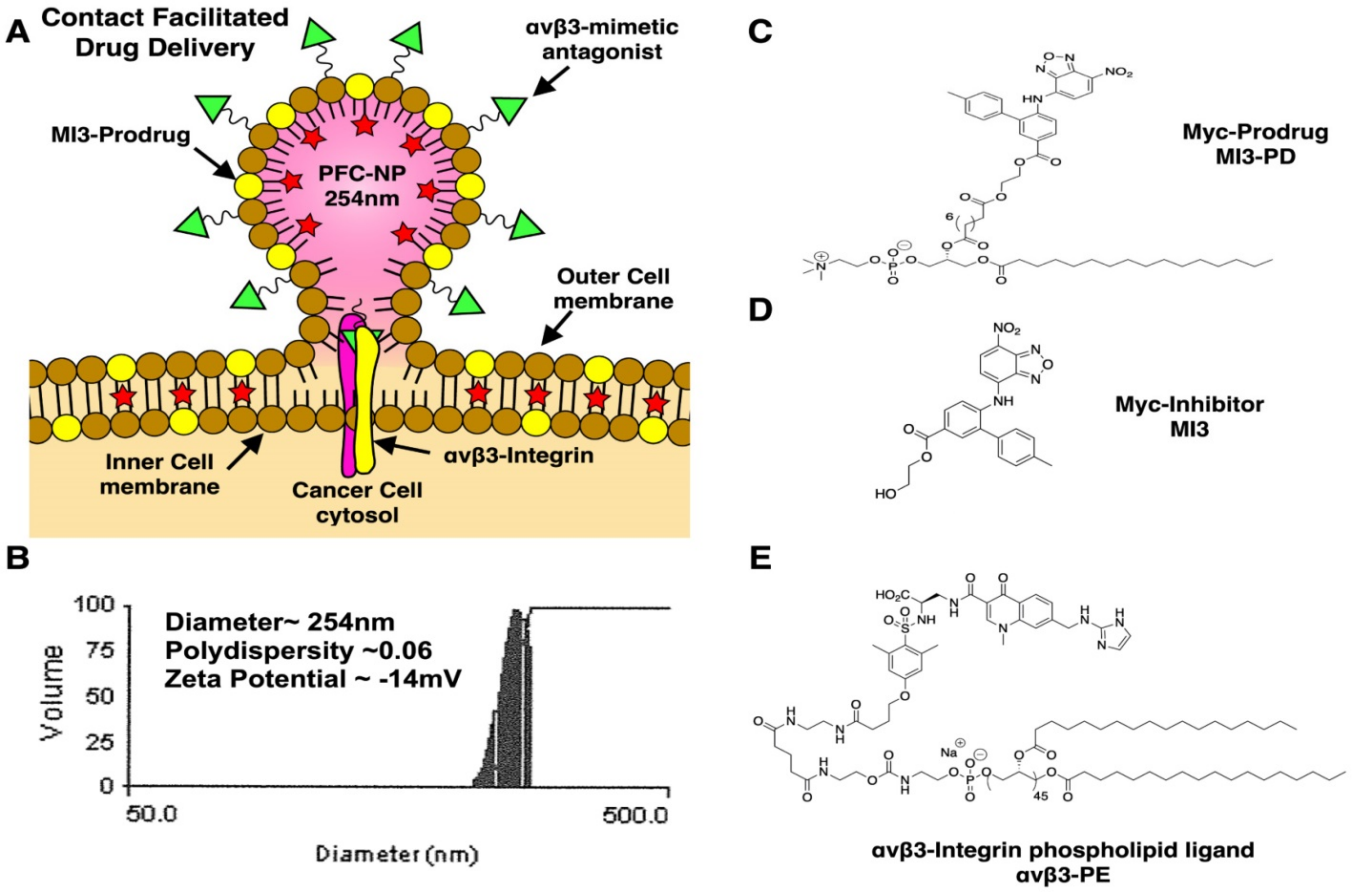
** Development of αvβ3 targeted MI3 prodrug perfluorocarbon nanoparticles (PFC). (A)** PFC nanoparticles deliver MI3 prodrug through a contact-facilitated drug delivery mechanism. **(B)** Analysis of the average PFC nanoparticle diameter, polydispersity and zeta potential **(C)** MYC/MAX dimerization inhibitor MI3 prodrug **(D)** MYC inhibitor MI3 **(E)** αvβ3-integrin targeting ligand.

αvβ3-targeted-MI3-PD perfluorocarbon NP were prepared as previously described and characterized 38 (see Supplemental Data for further discussion and Figure 1A). A microfluidized suspension of 20% (v/v) perfluorooctylbromide (PFOB, Exfluor Inc., Round Rock, TX, USA), 2.0% (w/v) of a surfactant co-mixture, and 1.7% (w/v) glycerin. The surfactant co-mixture of NP included: 0.15 mol% of α_v_β_3_-PEG2000-PE, 4 mol% of the MI3-PD, and the balance was high purity egg phosphatidylcholine (PC) (Lipoid LLC, Newark, NJ). The surfactant components were combined with the PFOB, deionized water, and glycerin. The mixture was pre-blended (Tissumizer Mark II, Tekmar, Cincinnati, Ohio, USA) then homogenized at 20,000 psi for 4 min (M110s, Microfluidics Inc., Westwood, MA, USA). Control αvβ3-targeted nanoparticles excluded MI3-PD. Routine NP characterization revealed: nominal size of 262 nm, polydispersity of 0.09, and zeta potential of -20 mV as shown in Figure 1B (Brookhaven Instruments Co, Holtsville, NY, USA). Transmission electron microscopy images of this nanoparticle were previously published 38.

### Synthesis of MI3-PD 2-Hydroxyethyl 4'-methyl-6-((7-nitrobenzo[c][1,2,5]oxadiazol-4-yl)amino)-[1,1'-biphenyl]-3-carboxylate (7jc28, MI3)

To a solution of 4'-methyl-6-((7-nitrobenzo[*c*][1,2,5]oxadiazol-4-yl)amino)-[1,1'-biphenyl]-3-carboxylic acid 39 (400 mg, 1.02 mmol, 1 eq) in anhydrous DMF (15 mL) was added 2-chloroethanol (76 µL, 1.12 mmol, 1.1 eq) and K_2_CO_3_ (211 mg, 1.53 mmol, 1.5 eq). The reaction mixture was stirred at 80 ºC for 3 h, at which point TLC indicated the reaction was complete. The reaction was cooled to room temperature then carefully acidified with 1M HCl. The mixture was extracted with ethyl acetate (EtOAc ×2), the organic layers were combined, washed with water (×3), brine and then dried over Na_2_SO_4_. The residue was re-suspended in ether, vigorously stirred for 30 min, then the product was isolated by vacuum filtration to afford the title compound as a bright red solid (376 mg, 85%): ^1^H (*d*_6_-DMSO) δ 2.26 (s, 3H), 3.75 (m, 2H), 4.35 (m, 2H), 5.00 (br, 1H), 6.25 (d, *J* = 8.8 Hz, 1H), 7.17 (d, *J* = 7.2 Hz, 2H), 7.35 (d, *J* = 7.2 Hz, 2H), 7.70 (d, *J* = 8.0 Hz, 1H), 8.11 (m, 2H), 8.43 (d, *J* = 8.8 Hz, 1H), 11.04 (s, 1H); ^13^C (*d*_6_-DMSO) δ 25.8, 64.2, 72.1, 108.0, 128.2, 133.3, 133.5, 134.4, 134.6, 134.7, 137.2, 139.7, 142.2, 142.5, 143.7, 144.4, 148.2, 149.2, 149.5, 170.3.

MI3 was used alone as the “free drug” or esterified with 1-palmitoyl-2-azelaoyl phosphatidylcholine (fatty acid modified oxidized lipid 16:0-9:0 COOH PC, PAzPC) through a dicyclohexyl carbodiimide (DCC)/4-dimethyl amino pyridine (DMAP) mediated coupling to produce MI3-PD (Figure 1C, D). The chemical structure of MI3-PD was characterized by MS spectrometric analysis (ESI-TOF (positive mode): *m/z* [C55H80N5O15P]^+^ Calculated. 082.22 Da.; Observed (M+H) 1083 Da.

### Characterization of the αvβ3-integring nonpeptide antagonist

The αvβ3-integrin ligand (αvβ3) was a quinolone nonpeptide antagonist (Figure 1E) developed by Bristol-Myers Squibb Medical Imaging (BMSMI, US patent 6,511,648 and related patents) that was initially reported and characterized as the ^111^In-DOTA conjugate RP748 and cyan 5.5 homologue TA145 40. The specificity of the αvβ3-ligand mirrors that of antibody LM609 as assessed by staining and flow cytometry, and it has a 15-fold preference for the Mn^2+^ activated receptor (21 nM) 41. The ligand has broad species cross-reactivity. Dissociation constants of the mimetic in humans established by BMSMI were: 1) αvβ3-receptor ELISA (biotin-Vn): <1 nM, 2) αvβ5 whole cell assay: 5.4 ± 1.9 µM, 3) αvβ5-receptor ELISA (biotin-Vn): 4 nM, 4) α5β1 whole cell assay: > 10 µM, 5) GP IlbIIIa PRP aggregation: > 10 µM, 6) isolated platelet GP IIbIIIa receptor: 5,289 µM 42. The peptidomimetic was coupled via a polyethylene glycol spacer (PEG2000) to phosphatidylethanolamine (αvβ3-PEG2000-PE, Gift from Kereos, Inc, St. Louis, MO). The targeted nanoparticles (NP) presented ~300 ligands/particle with an IC_50_ of 50 pM for the Mn^2+^-activated α_v_β_3_-integrin 43.

### Cell lines

The murine C57BL/6 PyMT-Bo1 luminal B breast cancer cell line (stably expressing GFP and firefly luciferase genes) was originally isolated from a transgenic MMTV-PyMT breast tumor, as previously validated and described 44. The PyMT-Bo1 *Itbg3* knockout cell line (PyMT-Bo1 β3 KO), was created by CRISPR genome editing as described below. The 4T1-GFP-FL cell line expresses green fluorescent protein (GFP) and firefly luciferase (FL) and was obtained from the David Piwnica-Worms lab 45. The MDA.MB.435 cell line was obtained from the ATCC. All cells were maintained at sub-confluence in DMEM with 10% fetal bovine serum (FBS) and 0.5% penicillin-streptomycin, in a humidified chamber under standard culture conditions. Low-passage stocks were used and regularly tested for Mycoplasma and maintenance of growth characteristics. Primary human umbilical vein endothelial cells (HUVEC) were obtained from Lonza (Basel, Switzerland), and express CD31, CD105, von Williebrand Factor VIII, and were positive for acetylated low-density lipoprotein uptake. HUVECs were grown to 70% confluence in VascuLife Endothelial Cell Culture Media (Lifeline Cell Technologies, Fredrick, MD, USA) and maintained for less than five passages.

### Generation of an integrin β3 (Itgb3) knockout breast cancer cell line

PyMT-Bo1 *Itbg3* knockout cells (PyMT-Bo1 β3 KO) were generated using the CRISPR/Cas9 system of genetic engineering. This work was done through the Genome Engineering and iPSC center (GEiC) at Washington University School of Medicine (St. Louis, MO), using the following guide RNAs (gRNAs):

SM903.itgb3.g4: CCTCAACAACGAGGTTATCCNGG;

SM903.itgb3.g13: CCGGGATAACCTCGTTGTTGNGG.

Genotyping by targeted deep sequencing of exon 9 of *Itgb3* showed a 25 base pair deletion in one *Itgb3* allele and a 31 base pair deletion in the other *Itgb3* allele. Flow cytometry analysis of integrin β3 demonstrates the complete loss of surface expression.

### Isolation and polarization of bone marrow macrophages

To generate primary macrophages, whole bone marrow was extracted from the femurs and tibias of mice and plated in petri dishes in MEM Alpha media containing 10% fetal bovine serum and 50 ng/mL M-CSF. For macrophage polarization assays, day 3 cultured macrophages were plated at 5x10^5^ cells per well in 6-well cell culture plates and treated for 6-48 h depending on the assay and polarized with IFN-γ (5 ng/mL) for M1 polarization or IL-4 (5 ng/mL) for M2 polarization or tumor-conditioned media. Tumor conditioned media was generated from the murine breast cancer cell line PyMT-Bo1. PyMT-Bo1 cells were cultured for 24-48 h. Harvested media was diluted in MEM Alpha macrophage media at a 1:2 ratio and M-CSF was maintained at 50 ng/mL.

### *In vitro* nanoparticle binding assay

Cells treated with 50 pM αvβ3-targeted NP, 50 pM non-targeted NP, or 50 fM of fluorescent 1 µm carboxylate-modified latex beads (L4655, Sigma-Aldrich). Cells were incubated for 3 h at the appropriate temperature (37 °C or 4 °C) with continuous shaking. After 3 h, each well was washed 3 times with PBS, and removed with 0.5% trypsin. Cells were analyzed by flow cytometry analysis on a BD LSRFortessa (BD Biosciences) and data analysis was performed with FlowJo software version 10.1 (Tree Star).

### RNAseq data acquisition and analysis

RNAseq normalized data were downloaded from the Gene Expression Omnibus database 46. Whole-tissue breast cancer RNAseq data were obtained from the GSE100925 as normalized FPKM 47. RNAseq data from breast cancer TAMs (CD45+CD3/56/19-CD11b+CD14+CD163+) and circulating monocytes (CD45^+^, CD3/CD19/CD56^-^, HLA-DR^+^) were downloaded from the GSE117970 dataset as normalized CPM (described in the original work) 47. Conversion of gene identifiers between entrez gene ID and official gene name was run on the Database for Annotation, Visualization and Integrated Discovery 48, 49 and verified on the individual gene's Uniprot entry. The corresponding gene list is shown in supplemental Table S1. Prism8 (GraphPad) was used to generate plots and calculate Pearson's correlation coefficient *r* and *p* values.

### Pharmacological inhibition of phagocytosis

For pharmacological inhibition of phagocytosis, macrophages were pretreated for 1 h with 5 μM of the phagocytic inhibitor Cytochalasin D (CytoD), (sc-201442, Santa Cruz Biotechnology), which inhibits actin polymerization on the plasma membrane. Alternatively, macrophages were pre-cooled at 4 °C for 1h, which also inhibits actin dynamics 50, 51. After 1 h, cells were then treated with either NP or latex phagocytic beads, at the appropriate culturing conditions (in the presence of CytoD, or at 4 °C).

### *In vitro* MI3-PD dosing

Macrophages were cultured for 3 days as described above. Cells were then trypsinized and plated onto petri dishes with MEM Alpha media and 50-100 ng/mL M-CSF and polarized with IFNγ (5 ng/mL), IL-4 (5 ng/mL) or tumor-conditioned media (1:2). The following day, cells were dosed with MI3 prodrug and fresh media containing M-CSF with cytokines or tumor conditioned media (TuCM). After 48 h cells were assessed by MTT or RNA was harvested with the RNeasy kit (Qiagen).

### MTT viability assay

The MTT viability assay was performed as described previously 52. For post-polarization assays, cytokine (IL-4, IFNγ, TuCM) was added 24 h prior to the addition of drug or nanoparticle. Pre-polarization assays were treated with drug or nanoparticle 1 h prior to the addition of polarizing cytokines or tumor conditioned media. Signal intensity is reported as OD570. Cell viability was assessed at 6-48 h.

### Real-time quantitative PCR (qPCR) analysis

Total RNA from cells was isolated with the RNeasy Mini Plus kit (Qiagen). Complementary DNA was made using the (qScript cDNA Synthesis Kit, Quanta). qPCR was performed using PerfeCTa SYBR Green FastMix (Quanta), with mouse-specific primers for mRNA genes of interest:* Actin*, *C-myc*, *Max*, *Akap12*, *Wnt5a*, *Maoa*, *Mrc1*, *Myc*, and *Pcsk5*, analyzed using the ∆∆Ct method. The primer sequences are shown in supplemental Table S2.

### Arginase 1 - yellow fluorescent protein (YFP) assay

Macrophages were isolated from mice genetically engineered to express the YFP protein downstream of the endogenous stop codon of the Arginase 1 gene (Jackson Labs, B6.129S4-*Arg1^tm1Lky^*/J) 53. Bone marrow macrophages were cultured as described, plated in MEM Alpha media containing 50 ng/mL M-CSF and 0.5 ng/mL of IL-4. After 24 h of polarization, cells were treated with MI3 prodrug for 48 h and *Arg1* expression read by YFP expression was assessed by flow cytometry.

### Animals Use

Animal studies were approved by and performed in accordance with the guidelines established by the Washington University Institutional Animal Care and Use Committee (IACUC). All mice were obtained from The Jackson Laboratory and were housed according to the guidelines of the Division of Comparative Medicine, Washington University School of Medicine.

### Murine cancer models and therapeutic dosing

To establish orthotopic mammary fat pad (MFP) tumors, 0.1×10^6^ PyMT-Bo1 β3 KO and 4T1-GFP-FL tumor cells were resuspended in 50 µL PBS and implanted into MFP tissue of 7-8 week-old female C57BL/6 mice. MI3-PD encapsulated in αvβ3-NP was dosed at 4.5 mg/kg per injection with 3 nanoparticle injections per experiment.

### Bioluminescence imaging (BLI) analysis

*In vivo* BLI was performed on IVIS50 (PerkinElmer, Waltham, MA) as previously described 44. Total photon flux (photons/sec) was measured from fixed regions of interest (ROIs) using Living Image 2.6. Investigators were blinded to treatment groups during all BLI analyses.

### Flow Cytometry of mammary fat pad tumors

Tumors were harvested, minced and incubated in 1X collagenase for 1 h at 37 °C shaking. Collagenased tumor was put through a 70 µm cell strainer, washed in media, and cell counted. Cells were prepped for flow cytometry by aliquoting 3 million cells into wells in a 96-well v-bottom plate. Cells were spun at 12000 RPM for 5 min at 4 °C, media removed and incubated for 20 min with Fc block. After FACS Buffer (PBS, 5% FBS, EDTA) wash, cells were incubated with antibody for 20 min, washed twice in FACS buffer and resuspended for flow cytometry on the BD X-20Fortessa. The antibody panel used for flow cytometry is available in supplemental Table S3.

### Statistical analysis

All data is shown as mean with error bars representing SEM. All sample sizes reported in the study are the minimum number of samples. For animal studies, sample sizes were estimated according to our previous experience. Statistical differences were analyzed using either a two-tailed *t*-test, ANOVA with Tukey's test for *a posteriori (*post-hoc) multiple comparisons or a two-tailed unpaired t-test with Bonferroni correction for *a priori* comparisons between a control group and experimental treatment groups of interest. Assumptions for ANOVA and t-test (independent samples, approximately normal distributions) for samples n>5 were sufficiently met, or used if a random sample of n≤5 were selected from an approximately normally distributed population. Non-normally distributed data were analyzed using a two-tailed Mann-Whitney *U*-test or a two-tailed Wilcoxon signed-rank test for matched-pairs. All tests were considered significant at *P*≤0.05*.* Data analyses were performed with Prism 8 (GraphPad Software).

## Results

### Integrin β3 is expressed on human breast cancer-associated macrophages and can be targeted by nanoparticles in mice

**Figure 2:**
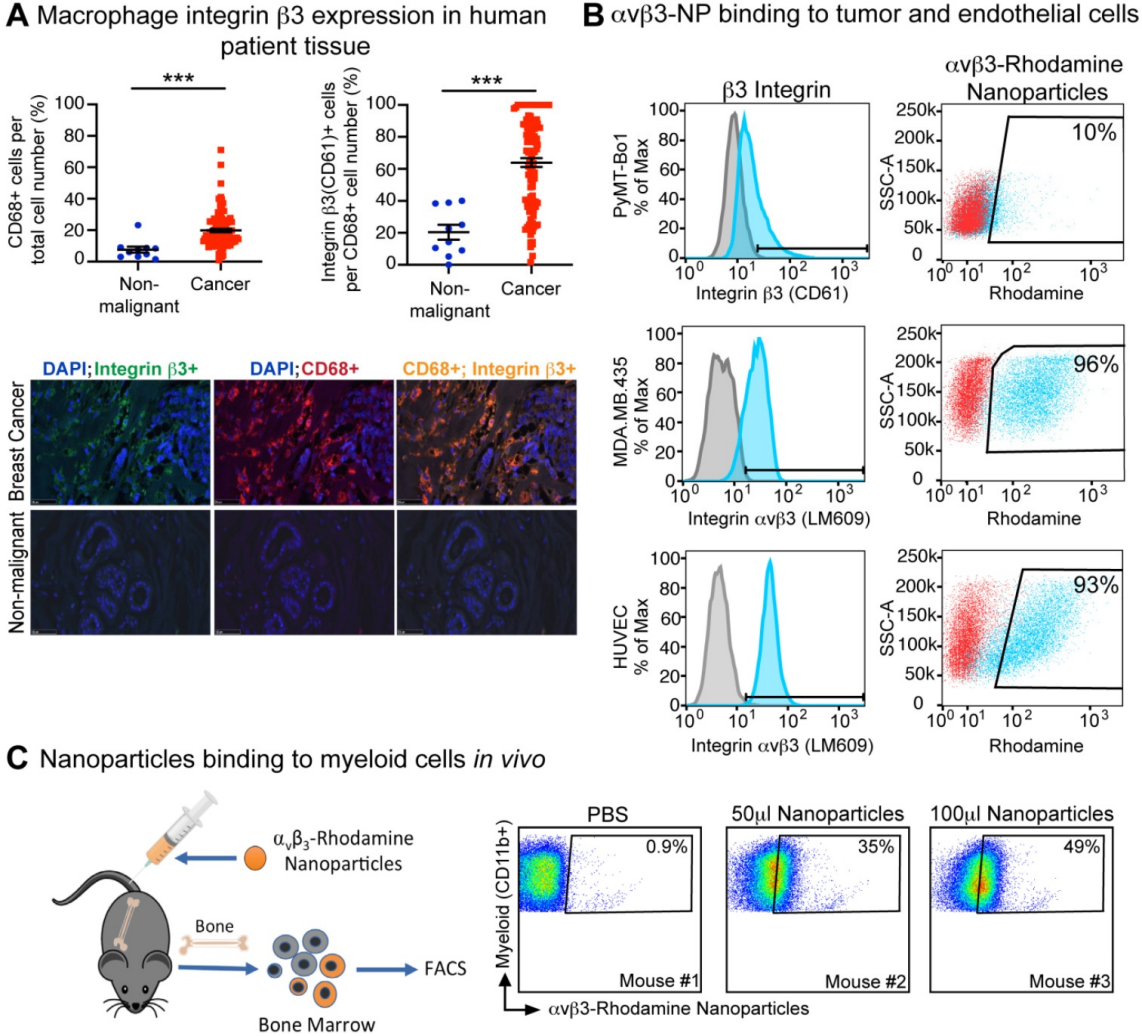
** Human breast cancer tumor-associated macrophages express β3 integrin. (A)** Immunofluorescence of CD68+ (n=10, n=108) and CD68+, CD61+ (n=10, n=106) macrophages in human non-malignant (blue) and malignant breast tissue (red), respectively (***P≤0.001). **(B)** Flow cytometry analysis of β3 integrin expression (blue) and nanoparticle uptake (rhodamine+) by murine breast cancer cell line (PyMT-Bo1, MFI 17), human melanoma (MDA.MB.435, MFI 27) and human endothelial (HUVEC, MFI 42) cell lines *in vitro*. Duplicate biologic replicates were preformed with representative images shown. **(C)** Flow cytometry analysis of bone marrow from PBS or αvβ3-rhodamine treated mice (n=3). MFI: median fluorescent intensity.

We have previously shown that integrin β3 is expressed on tumor-infiltrating M2 phenotype macrophages in mice 44. To determine whether integrin β3 is also expressed on breast cancer-associated macrophages, human tissue arrays of non-malignant and breast cancer tissues were assessed for infiltration of activated macrophage (CD68+) and expression of integrin β3 (CD61+). We found that the percent of CD68+ macrophages was significantly increased in human breast cancer compared to normal tissue. Further, 64% of CD68+ TAMs also expressed integrin β3 in breast cancer tissue as compared to 20% in normal breast tissue, indicating the potential to target these cells with αvβ3-NP-mediated drug delivery (Figure 2A).

Surface integrin β3 expression was evaluated by flow cytometry on tumor and endothelial cell lines using antibodies against murine integrin β3 (CD61) or activated human αvβ3 (LM609). As previously published, high levels of activated human αvβ3 were observed in MDA.MB.435 melanoma cells and HUVEC endothelial cells as indicated by the mean fluorescence intensity (MFI) of 42 and 27 respectively (Figure 2B). Integrin β3 expression was also observed in the murine estrogen receptor positive (ER+) breast cancer cell line PyMT-Bo1 but at relatively lower levels (17 MFI, Figure 2B). *In vitro* binding of αvβ3-NP labeled with rhodamine (αvβ3-rhodamine NP) revealed αvβ3-rhodamine NP uptake was similar to the amount of surface integrin expression observed by flow cytometry (CD61 or LM609, Figure 2B).

We have previously shown that αvβ3-NP bind to the tumor endothelium *in vivo* 54. We next assessed the ability of αvβ3-rhodamine NP to bind myeloid cells *in vivo*. Non-tumor bearing mice were injected with αvβ3-rhodamine NP or PBS. Bone marrow was harvested and analyzed by flow cytometry for myeloid cell marker CD11b and rhodamine fluorescence 3 h after inoculation. Rhodamine-positive CD11b+ cells were not detected in PBS treated mice (<1%) but were present in mice injected with 50 µl or 100 µl αvβ3-NP. Rhodamine+, CD11b+ bone marrow cells correlated with the amount of αvβ3-NP injected at 35% and 49% respectively (Figure 2C). These data indicate αvβ3-targeted nanoparticles can bind to a significant number of CD11b-expressing myeloid cells in bone marrow *in vivo*.

### Integrin αvβ3-targeted nanoparticles can deliver rhodamine payload to M2 macrophages in part through a phagocytosis-independent mechanism

**Figure 3:**
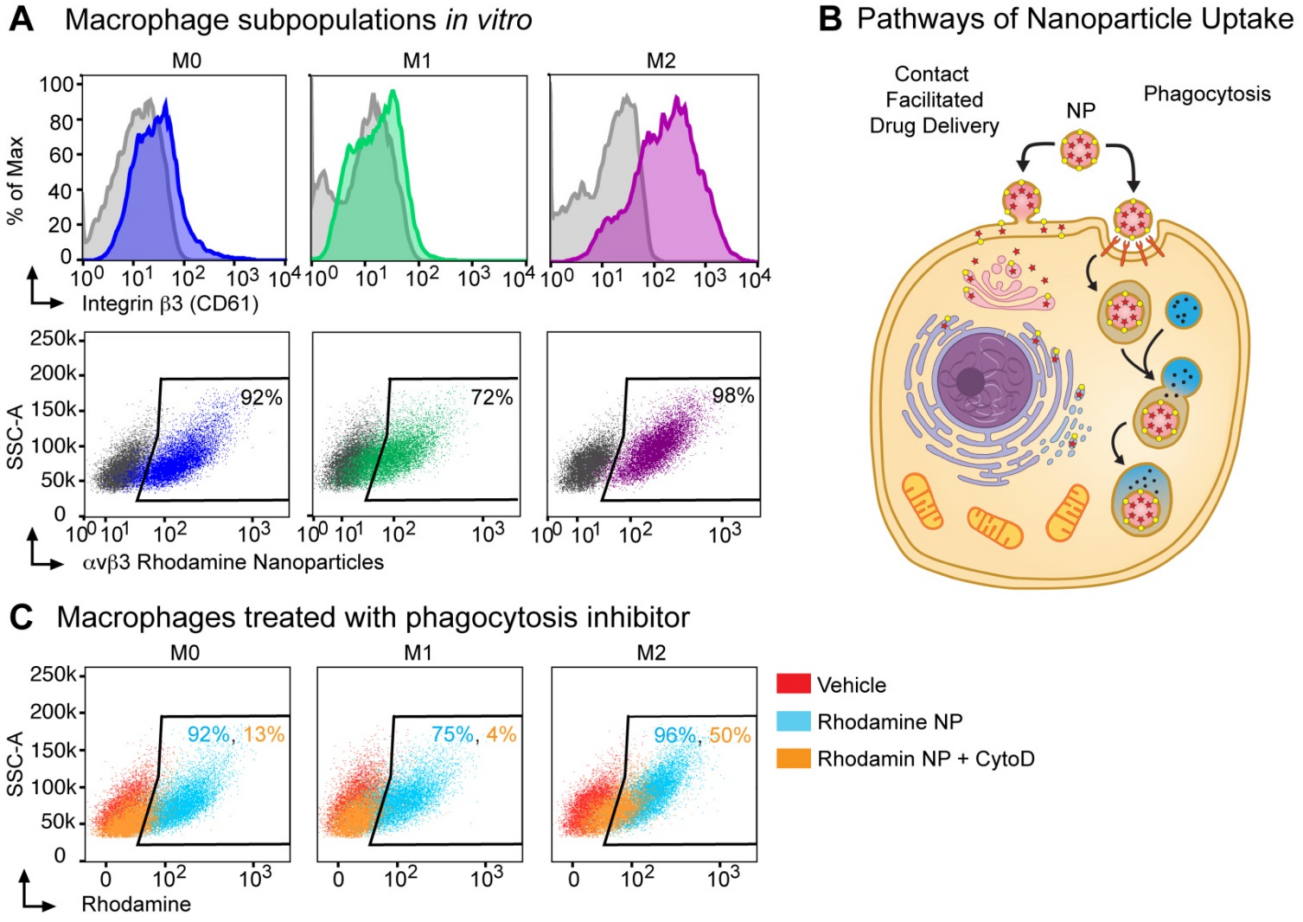
** Nanoparticle delivery of MI3 prodrug occurs in part through a non-phagocytic mechanism in M2 macrophages. (A)** Flow cytometry analysis for β3 integrin expression and the percent of cells positive for rhodamine following *in vitro* incubation with αvβ3 rhodamine labeled nanoparticles (NP) in M0 (blue, MFI 57), M1 (green, MFI 27) and M2 (purple, MFI 381) macrophage subsets. **(B)** Nanoparticles can deliver drug through a contact-mediated drug delivery mechanism or non-specific phagocytosis. Contact-facilitated drug delivery avoids sequestration and degradation in the endocytic pathway. **(C)** Flow cytometry analysis of the percent of cells positive for rhodamine in the presence (orange) or absence (blue) of phagocytosis inhibitor Cytochalasin D after *in vitro* incubation with αvβ3-rhodamine labeled nanoparticles as compared to vehicle treated controls (red). Representative experiments are shown. Biologic replicates were completed in triplicate for all experiments.

Next, we assessed integrin β3 expression on *in vitro* cultured bone marrow macrophage populations (BMMs): unpolarized macrophages (M0, M-CSF alone), M1 (classically polarized using M-CSF+IFNγ), M2 (alternatively polarized using M-CSF+IL-4). By flow cytometry, integrin β3 expression was increased 14-fold in M2 macrophages as compared to expression in M1 macrophages and 7-fold as compared to M0 macrophages (381, 27 and 57 MFI respectively, Figure 3A). To confirm that the αvβ3 targeting ligand used in these nanoparticles bind to M2 macrophages, we used an activated αvβ3 specific targeting ligand conjugated to a fluorescence probe and incubated with bone marrow macrophages isolated from WT or β3 deficient mice 55. The αvβ3 targeting probe showed a non-specific 4-fold increase in binding to β3 KO M2 macrophages compared to the unstained control while the binding was increased 12-fold in WT M2 macrophages (Figure S1). We next incubated bone marrow derived macrophages with αvβ3-rhodamine NP and found that greater than 70% of cells in all three subsets of macrophages (M0, M1 and M2) were rhodamine positive.

Typically, phagocytosis refers to the engulfment of particles of 500 nm of diameter or larger, achieved through reorganization of the actin cytoskeleton and lipid membrane. In professional phagocytic cells such as macrophages, this follows the activation of specific receptors, such as FcR. Macrophages can phagocytose polymeric particles, requiring strategies to prevent lysosomal degradation of cargo drugs. To assess whether αvβ3-rhodamine NP were taken up exclusively by phagocytosis or a non-phagocytic mechanism like contact facilitated drug delivery CFDD (Figure 3B) 37, 56-59, we evaluated uptake in the presence of phagocytosis inhibitors.

Cytochalasin D inhibits phagocytosis and macropinocytosis by disrupting actin polymerization. There was a marked cell number reduction in rhodamine uptake after incubation with αvβ3-rhodamine NP and cytochalasin D in the M1 macrophages of 4% and in the M0 macrophages of 13%; whereas, rhodamine cellular uptake by 50% was noted after incubation with αvβ3-rhodamine NP and cytochalasin D in the M2 macrophages (Figure 3C). These data suggest that M0, M1 and M2 macrophages can phagocytose αvβ3-rhodamine NP, however, of the macrophage subsets evaluated, the M2 macrophages, which have the highest αvβ3 expression, have the greatest ability to take up cargo through a phagocytosis-independent mechanism.

### MI3 prodrug reduces expression of MYC regulated genes with known roles in macrophages polarity

**Figure 4:**
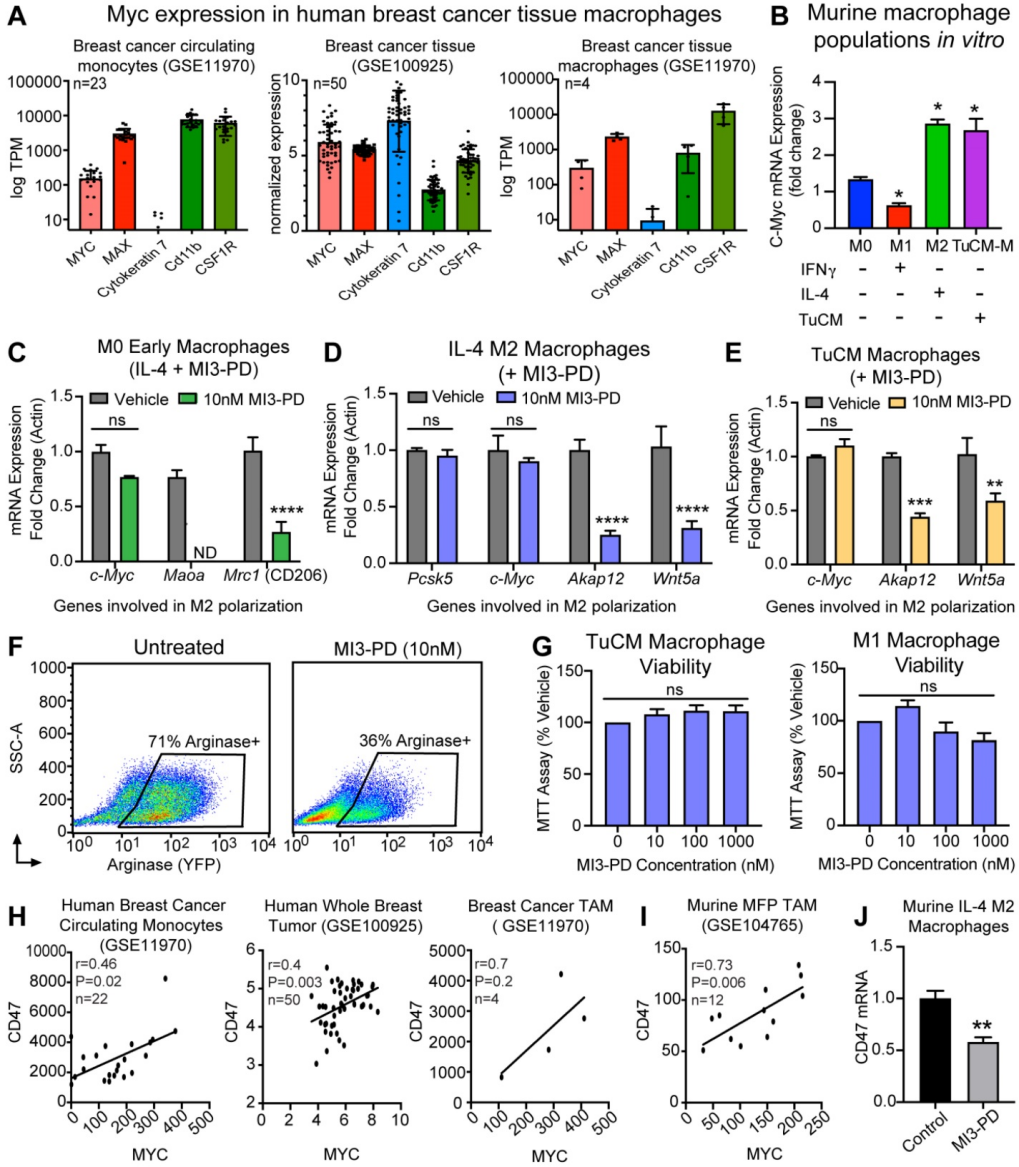
** MI3 prodrug treatment reduced expression of MYC regulated genes involved in alternative activation of M2 macrophages. (A**) Quantification of *MYC* and *MAX* expression in human breast cancer tissues from by RNAseq analysis. **(B)** qPCR of *Myc* mRNA expression in murine bone marrow macrophage subsets M0, M1 (IFNγ), M2 (IL-4) and tumor conditioned media (TuCM) polarized macrophages (6 h). Quantitative PCR of known MYC regulated genes in **(C)** M0 macrophages treated with IL-4 and MI3 prodrug (MI3-PD, 6 h), **(D)** established IL-4 M2 macrophages and **(E)** established tumor TuCM polarized macrophages treated with MI3-PD (24 h) post polarization. **(F)** Flow cytometry of *Arg1*-YFP reporter macrophages polarized with TuCM and treated with MI3-PD (48 h). **(G)** MTT viability assay for macrophages treated with MI3-PD (48 h). Data shown is the average of two biologic replicates. **(H)** Correlation of *CD47* and *MYC* expression in human breast cancer by *in silico* analysis of RNAseq data sets. **(I)** RNAseq data from murine breast cancer TAMs (DAPI^-^CD45^+^CD11b^+^Ly6G^-^Ly6C^-^F4/80^+^, GSE104765, n=12) was assessed and normalized values as presented in the original paper, were tested for correlation. **(J)** *Cd47* qPCR of murine M2 (IL-4) macrophages treated with MI3-PD (24 h). All qPCR experiments were shown as representative experiments and 2-3 biologic replicates were performed for each. P-values are denoted as follows: *P≤0.05, **P≤0.01, ***P≤0.001, ****P≤0.0001. ND: not detectable, ns: not significant.

*MYC* is an oncogene frequently expressed in cancer cells. *MYC* expression has more recently been reported in tumor-associated macrophages (TAMs) 60. To determine whether our targets *MYC* and *MAX* were expressed by macrophages in human breast cancer, publicly available RNAseq data sets on human breast cancer TAMs 47 were analyzed for *MYC* and *MAX* levels. *MYC* and *MAX* RNA were detected in circulating breast cancer monocytes (KRT7+, CD11b+, CSF1R+), whole breast cancer tissue (KRT7+, CD11b+, CSF1R+), and isolated breast cancer tumor infiltrating macrophages (KRT7 negative or low, CD11b+, CSF1R+), supporting a potential role for MYC in human TAMs (Figure 4A). Evaluation of murine BMM also demonstrated that *Myc* expression was upregulated upon IL-4-induced M2 polarization when normalized to M0 mRNA, whereas *Myc* expression was decreased by IFNγ-induced M1 polarization. Collectively, we found that M2 polarized BMMs (IL-4-induced, or tumor conditioned media induced) had increased expression of *Myc* mRNA as compared to M0 or IFNγ-induced M1 macrophages (Figure 4B).

To test the ability of our MYC-MAX dimerization inhibitor MI3 prodrug (MI3-PD) to reduce expression of known MYC-regulated genes involved in M2 polarization, mRNA expression of MI3-PD treated BMMs was assessed 60. MYC inhibition of early macrophages prior to IL-4-induced M2 polarization decreased known MYC regulated genes, including the M2 markers *Maoa* and CD206 (*Mrc1*) (Figure 4C). MI3-PD inhibition of more established M2 macrophages (after IL-4 polarization) significantly decreased expression of an alternative set of MYC regulated genes, including *Akap12* and *Wnt5a*. MI3-PD had no effect on expression of *Myc* or *Pcsk5*, a gene regulated independent of MYC (Figure 4D) 60. Finally, macrophages were polarized using breast cancer tumor-conditioned media (TuCM) to evaluate the effects of MYC inhibition on macrophages in a breast cancer context. MYC inhibition of BMM polarization with TuCM decreased *Akap12* and *Wnt5a*, an effect similar to what we observed in IL-4-polarized M2 macrophages treated with MI3-PD (Figure 4E).

We next assessed the ability of MI3-PD to reduce protein expression of well-known M2 polarity marker Arginase I (ARG1). The transgenic B6.129S4-*Arg1^tm1Lky^*/J mice (ARG1-YFP) express yellow fluorescent protein (YFP) downstream of the *Arg1* gene, reporting *in vivo* on the level of ARG1 biosynthesis. To validate in live cells the effect of MYC inhibition, BMM were isolated from ARG1-YFP, cultured and polarized with TuCM *in vitro*, and then treated with MI3-PD 53. MI3-PD inhibition reduced arginase expression by 35%, compared to vehicle treated M2 BMMs (Figure 4F). Interestingly, MI3-PD had no effect on macrophage viability in TuCM polarized macrophages, or IFNγ-polarized M1 macrophages *in vitro* (Figure 4G).

CD47 is a key receptor necessary for cells to avoid the immune system, often referred to as the “don't eat me” signal. Recent work has shown that MYC can regulate *CD47* expression in tumor cells, and that anti-CD47 antibody treatment of tumors increases the number of M1 macrophages in the tumor microenvironment 61, 62. We found through *in silico* assessment of RNAseq data sets that *CD47* expression correlated with *MYC* expression in human breast cancer circulating monocytes, whole breast tumors, human breast cancer TAMs 47, and mouse mammary fat pad tumor TAMs 63 (Figure 4H,I). Notably, treatment of M2 macrophages with MI3-PD significantly reduced *CD47* expression as compared to vehicle treated cells (Figure 4J). Taken together, these data show that MI3-PD treatment reduced expression of MYC-regulated genes in protumor M2 macrophages *in vitro*.

### Integrin αvβ3-targeted MI3-PD nanoparticles reduce mammary fat pad tumor-associated macrophages *in vivo*

**Figure 5:**
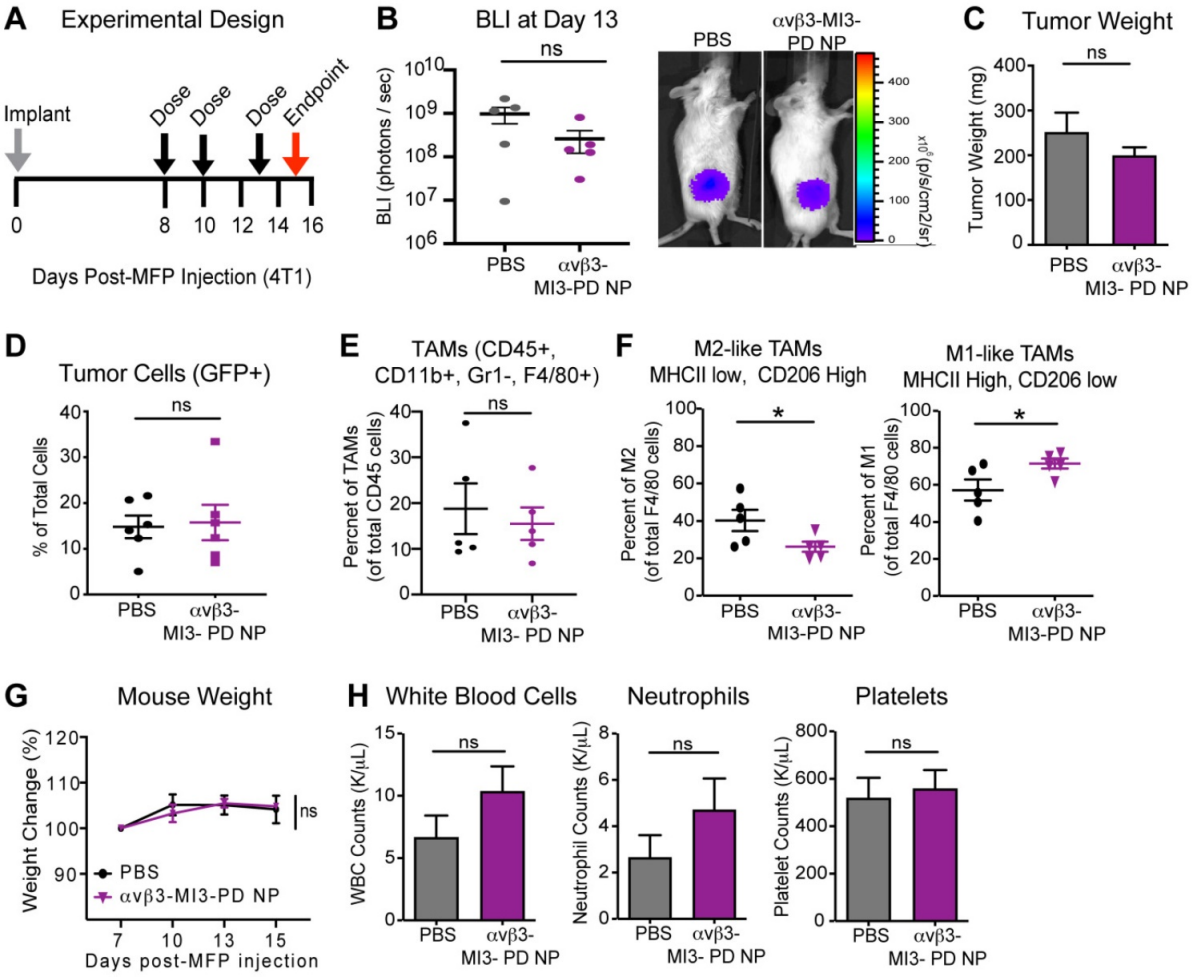
** αvβ3-targeted MYC inhibitor MI3 loaded nanoparticles decreased M2 tumor-associated macrophages in the 4T1-GFP-FL (4T1) murine breast cancer model. (A)** 4T1 cells were implanted into the mammary fat pad (MFP, day 0) and mice were given three doses of PBS or αvβ3-MI3-PD nanoparticles (NP, n=6/group). **(B)** Bioluminescence imaging (BLI) of *in vivo* tumor growth (*P≤0.05) **(C)** Tumor weight at study endpoint (day 16, n=5 mice/group). Flow Cytometry of **(D)** GFP+ tumor cells, **(E)** Tumor-associated macrophages (TAMs) per CD45+ cells and **(F)** M2-like and M1-like macrophages per total TAMs, isolated from MFP tumors at day 16 (n=5/group, *P≤0.05). **(G)** Mouse weight during treatment. **(H)** Complete blood counts at study endpoint. NP: nanoparticle; WBC: white blood cell; ns: not significant.

When applied systemically, MYC inhibitors are quickly degraded in blood, leading to reduced drug bioavailability 36, 64. Encapsulation within nanoparticles could increase efficacy by enhancing stability in circulation, increasing the amount of drug membrane payload delivered directly to target cells, and reducing off-target effects and toxicity. To evaluate whether αvβ3-MI3 PD nanoparticles were effective *in vivo*, mice bearing mammary fat pad (MFP) tumors were established, using an aggressive triple-negative murine breast cancer line 4T1-GFP-FL (BALB/C) that expresses integrin αvβ3 (Figure S2). Mice were given three doses of PBS or αvβ3-MI3-PD NP and tumors were evaluated by flow cytometry at study endpoint. Early stage tumors were assessed due to the large number of TAMs and the presence of both M1 and M2 macrophage subsets, compared to later stage tumors where M2 subsets predominate. We first assessed the effect of αvβ3-MI3-PD NP treatment on tumor growth by bioluminescent imaging (BLI) and tumor weight. Tumor growth at the early time point as measured by BLI (Figure 5B) and tumor weight at endpoint (Figure 5C) was similar between PBS and αvβ3-MI3-PD NP treatment groups. Following collagenase digestion, tumor composition was evaluated by flow cytometry. Analysis of tumor cells (GFP) showed there was no significant difference between control and treatment groups (Figure 5D). The percent of tumor-associated macrophages (TAMs: CD45+, CD11b+, GR1-, F4/80+) per total CD45+ cells within the MFP tumors was similar between treatment groups (Figure 5E). However, the percent of protumor M2 TAMs (CD45+, CD11b+, GR1-, F4/80+, MHCII low, CD206 high) was significantly decreased (14%) in αvβ3-MI3-PD NP treated mice, as compared to PBS (Figure 5F). Moreover, the percentage of antitumor M1 TAMs (CD45, CD11b, GR1-, F4/80+, MHCII high, CD206 low) was significantly increased (14%) in MFP tumors of αvβ3-MI3-PD NP compared to PBS treated mice (Figure 5F, Figure S3). For toxicity assessment, there were no differences between groups in the percent change in body weight, and for complete blood counts (CBC), including white blood cells, neutrophils, and platelets (Figure 5G,H). These data suggest that αvβ3-MI3-PD NP changed the composition of breast cancer tumor-associated macrophages, biasing towards low M2 TAMs, while inducing little systemic toxicity.

### αvβ3-MI3-PD nanoparticles do not directly target tumor cells to decrease M2 macrophages in breast tumors

**Figure 6:**
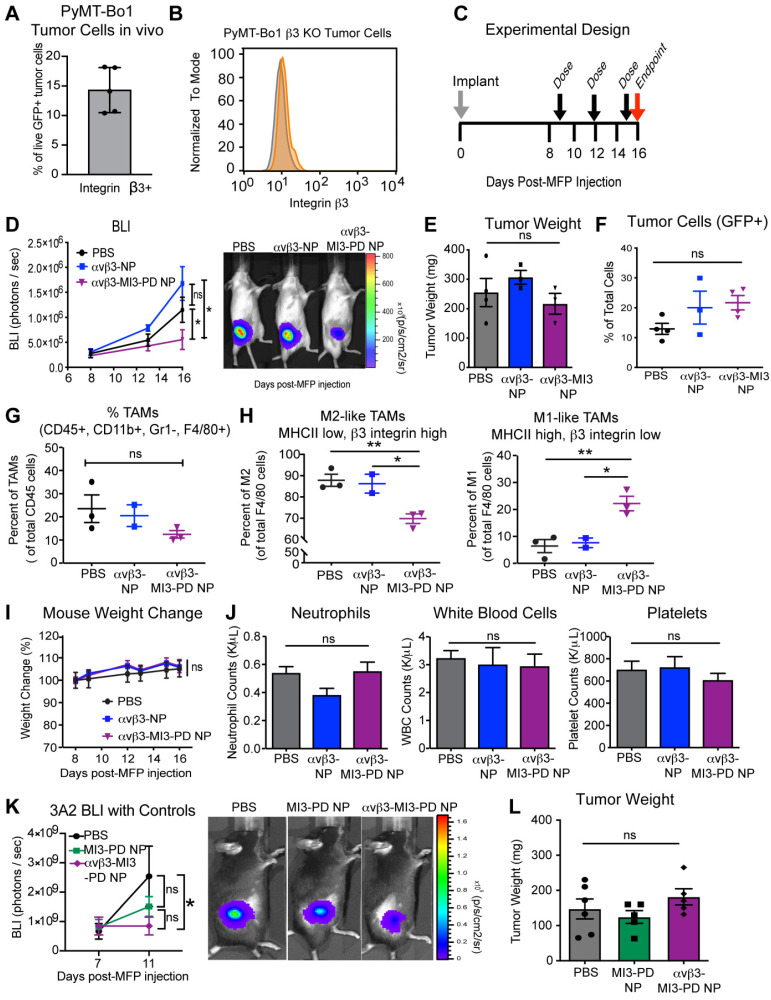
** MYC inhibitor MI3 reduces pro-tumoral M2-like macrophages and enhances M1-like macrophages in an *in vivo* breast cancer model. (A)** Integrin β3 expression on PyMT-Bo1 tumor cells isolated from breast cancer metastases in bone. **(B)** Flow cytometry of β3 expression (orange) **(C)** β3 KO cells were implanted into the mammary fat pad (MFP) of mice that were treated with PBS, no drug αvβ3-nanoparticles (NP) or αvβ3-MI3 prodrug nanoparticles (αvβ3-MI3-PD NP, n=4/group). **(D)** Bioluminescence imaging (BLI) of *in vivo* tumor growth (*P≤0.05). **(E)** Tumor weight at day 16. Flow cytometry analysis of **(F)** GFP+ tumor cells **(G)** Tumor associated macrophages (TAMs: CD45+, CD11b+, Gr1-, F4/80+) per CD45+ cells and **(H)** M2-like (MHCII low, β3 integrin high) and M1-like macrophages (MHCII high, β3 integrin low) per TAMs (n=2-3 mice/group) in day 16 MFP tumors. **(I)** Mouse weight during treatment. **(J)** Complete blood counts at study endpoint (n=3-4/group). (K) BLI of *in vivo* tumor burden (n=5/group). (L) Tumor weight at day 15. *P≤0.05, **P≤0.01, ns: not significant; WBC: white blood cell.

The reduction in M2 macrophages in our 4T1 breast tumors treated with αvβ3-MI3-PD NP could be explained, theoretically, either by direct effects on αvβ3+ macrophages by MI3, as shown *in vitro*, or by indirect effects of MYC inhibition in the αvβ3+ 4T1 cancer cells. In addition the 4T1 model has a lower M2/M1 ratio compared to the PyMT breast cancer models, which could underestimate the effect of M2 targeted agents. Therefore, we next evaluated αvβ3 directed MI3-PD NP therapy in a second breast cancer model, PyMT Bo1, which we engineered to lack tumoral β3. We selected PyMT-Bo1, which is a C57B/6 syngeneic ER+ luminal B type breast cancer cell line that expresses αvβ3 *in vivo* (Figure 6A) and has a higher M2/M1 ratio than 4T1 cells *in vivo*. We used the CRISPR/Cas9 system to genetically disrupt β3 expression (Figure 6B) and established PyMT-Bo1 β3 knockout (KO) MFP tumors and treated with the same dosing strategy as the 4T1 model (Figure 6C). We selected an ER+ luminal B type breast cancer cell line PyMT-Bo1 as a second immunocompetent breast cancer model. Although PyMT-Bo1 cells express relatively low levels of β3 integrin compared to endothelial cells (Figure 2B), we have observed upregulation of β3 expression *in vivo* (Figure 6A, Figure S4A) and therefore used the CRISPR/Cas9 system to genetically disrupt β3 expression (Figure 6B). We established PyMT-Bo1 β3 knockout (KO) MFP tumors and treated with the same dosing strategy as the 4T1 model (Figure 6C). To demonstrate that macrophage effects were specific to MI3-PD-mediated MYC inhibition, rather than due to non-specific nanoparticle-binding effects, mice were treated with PBS, drug-free αvβ3-NP, or αvβ3-MI3-PD NP. In this experiment, bioluminescent imaging (BLI) was used to assess the presence of cancer cells expressing firefly luciferase, as opposed to non-luciferase expressing host cells, within the tumor. By this measure, tumor burden significantly decreased with αvβ3-MI3-PD NP therapy, as compared to PBS or αvβ3-NP treated control groups (Figure 6D). Tumor weight was not changed between treatment groups (Figure 6E). The similar tumor size could be due to an influx of immune or other cell types within the αvβ3-MI3 NP treated tumor or because tumors were harvested at early stages.

We further evaluated tumor composition by flow cytometry and found no significant difference in the number of tumor cells as a percent of total cell number (Figure 6F). As with the 4T1 experiments, the overall number of TAMs (CD45+, CD11b+, GR1-, F4/80+) per CD45+ cells was not different between treatment groups (Figure 6G), but the composition of macrophage subsets were. There was a significant decrease (18%) in the percent of M2 TAMs (CD45+, CD11b+, GR1-, F4/80+, MHCII low, β3 integrin high) and a significant increase (16%) in the percent of M1 TAMs (CD45+, CD11b+, GR1-, F4/80+, MHCII high, β3 integrin low) with αvβ3-MI3-PD NP therapy (Figure 6H, Figure S4B). There was no change in M2 and M1 ratios in the PBS and αvβ3-NP control groups, indicating this effect was not due to the nanoparticle components or the targeting ligand (Figure 6H). Further, there was no change in MDSC or T cell populations with αvβ3-MI3-PD NP treatment (Figure S5). The toxicity assessments of percent change in mouse weight and blood counts, circulating neutrophils, white blood cells, or platelets remained the same between treatment groups αvβ3-MI3-PD NP treatment, as compared to the controls (Figure 6I, J).

To evaluate whether non-targeted MI3-PD nanoparticles (MI3-PD NP) could reduce tumor burden, we treated mice with PyMT-Bo1 β3 KO MFP tumors with PBS, MI3-PD NP or αvβ3-MI3-PD NP. Tumor burden was reduced (P<0.05) in the targeted MI3-PD nanoparticle group but not decreased (P>0.05) with non-targeted MI3-PD NP, suggesting the reduction in tumor burden as assessed by luciferase expression in tumor cells was a result of targeted delivery of MI3 prodrug (Figure 6K). Tumor weight was unchanged between treatment groups similar to our prior results in the 4T1 and PyMT experiments (Figure 6L). Tumor weight is comprised of a dynamically changing mixture of inflammatory cells, edema, fibrosis, and necrosis as well as malignant cells, which themselves occupy only a fraction of the volume 65. Together, these data suggest that down modulation of protumor M2 macrophages by αvβ3-MI3-PD NP decreased (P<0.05) tumor activity in both an ER+ a triple negative breast cancer model two mouse genetic backgrounds without any hematological toxicity.

## Discussion

Tumor-associated macrophages (TAMs) promote tumor growth and metastasis while also reducing the effectiveness of chemotherapy and immunotherapy. We and others have shown that murine protumor M2 macrophages have increased MYC and integrin αvβ3 expression. We reported that integrin αvβ3-expressing M2 macrophages promote breast cancer tumor growth in preclinical models 44, so we proposed a two-step therapeutic approach using a small molecule inhibitor to MYC-MAX by a vitronectin peptidomimetic targeting antagonist to the activated form of integrin αvβ3. We show for the first time that TAMs from human patient breast cancers express integrin αvβ3 and they also express MYC. Although MYC is an important target in tumor cells, MYC has not been therapeutically targeted in tumor-associated macrophages *in vivo*. We found that MYC inhibitor MI3, delivered through αvβ3-targeted NP, reduced M2 macrophages while preserving beneficial M1 macrophages *in vivo*. These effects were observed in two mouse models of ER+ and triple negative breast cancer in two animal backgrounds. *MYC* is expressed in human and mouse macrophages cultured *in vitro*, but *MYC* expression in breast cancer TAMs has not been explored. TAM populations often consist of a mixture of tissue resident macrophages and recruited monocytes. By RNAseq analysis of published data sets, we found that circulating monocytes and tumor-associated macrophages from breast cancer patients express *MYC*. Macrophages cultured in tumor conditioned-media also upregulated *Myc*, at similar levels to those induced during M2-polarization by IL-4. These data provide new support for the role of *MYC* in breast cancer TAMs, and highlight the therapeutic potential of targeted drug delivery against protumor M2 TAMs. Interestingly, *MYC* expression in tumor cells also promotes breast cancer progression, indicating that anti-MYC therapy could have compounding antitumor benefits, which are currently under investigation.

MYC can act as an amplifier of multiple signaling pathways, regulating the expression of a number of genes, which can be used as a functional marker for MYC/MAX activity. Treatment of murine peritoneal macrophages and human monocytes with MYC/MAX inhibitor 10058-F4 (MI1) *in vitro* decreased genes known to be involved in alternative M2 polarization 60. Some studies have suggested that the differences in the tissue origin of TAMs populations may result in different functions while others, indicate microenvironmental cues educate these TAMs towards a similar phenotype 66. We therefore investigated the role of MYC in bone marrow macrophages (BMM), polarized towards an M2 phenotype with IL-4 or with media conditioned by a breast cancer cell line. We found that treatment of BMM with MI3 prodrug, which has a longer intracellular half-life than previous MYC inhibitor MI1 39, 67, inhibited a similar set of genes important in M2 polarization (*Maoa*, *Akap12*, *Wnt5a*, and *Mrc1*). Moreover, we show new data indicating that the timing of MYC inhibition relative to addition of the polarizing stimulus can affect the transcription of different sets of MYC target genes. Downregulation of the key markers of M2 polarization, *Cd206* and arginase 1, suggests that nanoparticle-mediated drug delivery of MI3 MYC inhibitors could reduce M2 macrophage polarization and function *in vivo* 68.

Furthermore, we found that MYC inhibition can decrease *Cd47* in tumor-associated macrophages *in vitro*. CD47 has emerged as an important checkpoint for the immune system 69. Tumor cells upregulate *CD47*, known as the “don't eat me” signal, to escape recognition and clearance by immune cells including macrophages. MYC has been shown to regulate CD47 expression in some tumor types 61. Inhibition of CD47 on tumor cells promotes M1 macrophages in the tumor microenvironment, although it is unclear whether this occurs through increased recruitment of M1 macrophages or reprogramming of existing macrophages toward an M1 phenotype 62. We also found that *MYC* expression correlates with *CD47* expression in human whole breast tumor tissue, circulating monocytes, and tumor-associated monocytes in patients with breast cancer, indicating that MYC regulation of *CD47* may be conserved within breast cancer TAMs as well. We hypothesize that MYC downregulation of *Cd47* may contribute to the M1/M2 shift in our models of breast cancer. Additional work will be needed to investigate this mechanism.

Direct macrophage targeting with nanotechnologies, has focused on exploitation of the surface macrophage activation markers CD44 and CD206, which have a higher expression on M2 but are also expressed on M1-type cells 70-73. Our group has previously shown that integrin αvβ3 is not expressed at high levels on unpolarized myeloid cells or M1 macrophages, but is upregulated on M2 macrophages; furthermore, we found that integrin β3 plays a functional role in maintaining macrophage homeostasis and serves as a negative inhibitor for M2 function 44. Here, we show that integrin β3 is expressed on the majority of TAMs in human breast cancer patient tissues and we use αvβ3-targeted nanoparticle technology to deliver our MI3 prodrug *in vivo*.

To demonstrate NP targeting of MYC in M2 macrophages while preserving M1 macrophages, we evaluated the effects of our NPs using small early stage tumors, which in our hands, have a large immune component including both M1 and M2 macrophages. We found that αvβ3-MI3-NP significantly decreased M2 macrophages and increased M1 macrophage numbers in two breast cancer models. Tumor weight was not changed at the time of analysis although changes in bioluminescent activity were observed. We demonstrated a significant decrease in tumor burden by BLI with αvβ3-MI3-PD treatment. This reduction was not present in the αvβ3-NP or MI3-PD nanoparticles control groups. There are several challenges to evaluating tumor burden as tumor weight may not reflect changes in the cellular composition of a tumor. Bioluminescence imaging represents tumor activity *in vivo* without mechanical tissue disruption and may be a better reflection of real time tumoral changes; however BLI depends on ATP, oxygen and luciferin availability. Interestingly, there is clinical evidence that immune therapies often change the tumor cellular composition and metabolic uptake as measured by FDG PET, before eventually reducing tumor size 74. Radiology societies have modified the criteria for evaluating treatment response in solid tumors to account for unchanged or pseudo-progression of tumor volumes due to the influx of inflammatory cells 75. Clinical studies have shown that immune checkpoint inhibitors are less effective in some breast cancer subtypes like ER+ cancers. It is thought that macrophages play a large functional role in immune suppression in breast cancer. Clinical testing of generalized macrophage inhibitors are underway but there are concerns for inhibiting anti-tumor M1 phenotype macrophages as well. Our data suggest that patients with higher levels of TAMs could derive more benefit from immune checkpoint therapies when combined with macrophage-targeted therapies such as αvβ3-MI3-PD NP. It is possible that αvβ3-MI3-PD NPs had less impact on tumor cell burden as measured by BLI in the 4T1 triple negative breast cancer compared with the ER+ PyMT-Bo1 breast cancer, because there are significantly lower percent of M2-type TAMs in the 4T1 model as compared to the PyMT-Bo1 control tumors.

In macrophages, activation of specific receptors is required for phagocytosis and includes opsonin, scavenger and toll like receptors, among others 76. Receptor expression can be specific to macrophage subtype and determines the type of particles (bacteria, parasites, cell debris) phagocytosed 77, 78. Uptake of diverse particles by specific receptors, suggests M1 and M2 macrophages could use different internal mechanisms to process phagocytic particles and differences in phagosome maturation (ie. pH changes and pathway kinetics). Recent work has shown M2 macrophages may have increased phagosome maturation 79, however; for the purpose of this work, we did not explore downstream processes but instead used a broad microtubule inhibitor (Cytochalasin D) to block actin polymerization dependent phagocytosis and macropinocytosis. Control studies using agarose beads showed complete inhibition of bead uptake in M2 macrophages following CytoD treatment indicating CytoD concentrations were sufficient to block phagocytosis. Some studies have shown inhibitors can affect other endocytic pathways and we cannot rule out inhibition of clatherin-mediated endocytosis in addition to phagocytosis. However, several papers have demonstrated that CytoD treatment in macrophages, completely inhibit phagocytosis, but does not affect other endocytic pathways 80-82. These data in combination with our *in vivo* reduction of M2 macrophages suggests αvβ3 targeted NP can deliver drug to M2 macrophages that avoids degradation in the phagocytic pathway, resulting in more efficacious concentrations of drug in the cytosol.

A key finding of this work was the observation that integrin β3 is expressed on human breast cancer tumor-associated macrophages. In this paper we used tumor cell lines expressing β3 or β3 KO tumor cell lines to examine the effects of αvβ3-MI3-PD nanotherapy on β3-expressing macrophages. Because triple-negative breast cancers express elevated levels of MYC, we speculate that future studies developing similar tumor cell lines may help explore this dual targeting strategy in this group of patients, who have limited options for targeted therapies.

Many other cell types in the tumor microenvironment express αvβ3 integrin including: tumor induced angiogenic cells, bone-residing osteoclasts, immune cells, and some types of tumor cells which could be targeted by the αvβ3-MI3-PD NP 83. However, the integrin αvβ3-targeting peptidomimetic binds with high affinity to the ligand-binding domain exposed with activated integrins, avoiding binding to inactivated αvβ3 integrin on quiescent cells. We have previously shown that integrin αvβ3-targeting to the neovasculature can be used to specifically deliver cargoes with αvβ3-PFC NP in pathologic animal models of cardiovascular, inflammatory disease and cancer 54, 84, 85. The cMYC-MAX pathway is upregulated in tumor neovasculature, which also expresses the activated integrin 86. αvβ3-fumagillin NP target targeting neovascular endothelial induce apoptosis releasing nitric oxide that can suppress macrophage inflammatory responses 84. Importantly, αvβ3 is only expressed on newly forming blood vessels and not the established tumor vasculature. Thus, the positive anti-tumor response observed with αvβ3-MI3-PD NP may reflect a complicated anti-tumor response.

Our data show that cMYC-MAX is an opportune therapeutic target for manipulating the TAM population away from tumor-promoting macrophages and that a small molecule antagonist, modified into a phosphatidylcholine prodrug, protected the compound from metabolism during circulation and allowed a unique αvβ3 NP delivery mechanism (CFDD) to circumvent enzymatic degradation within the phagocytosis pathway and discharge directly into the intracellular membranes. Future research will need to refine and optimize this concept with a focus on corroborative immunohistological analysis, longer treatment courses, treatment of larger tumors, and evaluation in metastatic models. Additionally, the further use of CRISPR/Cas9 to disrupt integrin β3 expression in 4T1 breast cancer line could establish a useful breast cancer model to further evaluate whether αvβ3-MI3-PD NPs are effective against tumor cells *in vivo*, and the use of a smaller 20nm nanoparticle, similar to that we reported in multiple myeloma models 67, may enhance tumor penetration and effective extravasation. Finally, we propose to investigate whether macrophage repolarization using αvβ3-MI3-PD NPs can enhance responses to immune checkpoint inhibitors in breast cancer models.

In summary, this research demonstrates the potential of MYC-MAX inhibition with a small molecule to affect specific changes in the tumor promoting M2 macrophage population. A free small molecule inhibitor known to have poor stability in circulation was modified into a lipid prodrug and incorporated into the phospholipid surfactant of targeted perfluorocarbon nanoparticles. MYC inhibition *in vitro* decreased markers of M2 polarization while αvβ3-mediated drug delivery of the MYC inhibitor MI3-PD, decreased numbers of M2 TAMs without decreasing M1 macrophages in mouse models of ER+ and triple-negative breast cancer. Moreover, BLI of these breast cancer models demonstrated significant reductions in tumor cells following αvβ3-MI3-PD NP treatment. The overarching conclusion of this research is that cMYC-MAX inhibition is an important mechanistic target for anti-tumor treatment, particularly regarding the TAM population relative polarization, which is enabled by αvβ3-targeted nanotherapy.

## Supplementary Material

Supplementary figures and tables.

## Abbreviations

ANOVA: analysis of variance
ATCC: American type culture collection
b-HLHZIP: basic helix-loop-helix leucine zipper
BLI: bioluminescence imaging
BMM: bone marrow macrophages
CFDD: contact facilitated drug delivery
CRISPR: clustered regularly interspaced short palindromic repeats
CytoD: cytochalasin
DMEM: Dulbecco's modified eagle medium
ER+: estrogen receptor positive
FACS: fluorescence-activated cell sorting
FBS: fetal bovine serum
FL: firefly luciferase
GFP: green fluorescent protein
gRNA: guide RNA
HUVEC: human umbilical vein endothelial cells
IACUC: institutional animal care and use committee
IFN-λ: interferon gamma
IL-4: interleukin 4
KO: knockout
M-CSF: macrophage colony-stimulating factor
MDSC: myeloid derived suppressor cell
MEM Alpha: minimum essential medium
MFI: median fluorescence intensity
MFP: mammary fat pad
MI3: myc inhibitor 3 [2-Hydroxyethyl 4'-methyl-6-((7-nitrobenzo[c][1,2,5]oxadiazol-4-yl)amino)-[1,1'-biphenyl]-3-carboxylate]
MMTV-PyMT: mouse mammary tumor virus - polyomavirus middle T-antigen
MPS: macrophage monocyte phagocytic system
MTT: 3-(4,5-dimethylthiazol-2-yl)-2,5-diphenyltetrazolium bromide
NP: nanoparticle
PBS: phosphate buffered saline
PD: prodrug
PFC: perfluorocarbon
RPM: revolutions per minute
SEM: standard error of the mean
TAM: tumor-associated macrophages
TuCM: tumor conditioned media
αvβ3: integrin heterodimer of subunits αv and β3
αvβ3-NP: αvβ3 targeted nanoparticles
