# Supplementary material for: Nanotherapy delivery of c-myc inhibitor targets Protumor Macrophages and preserves Antitumor Macrophages in Breast Cancer
Source: Theranostics. 2020 Jun 12;10(17):7510–26. doi: 10.7150/thno.44523 (PMC7359087; doi:10.7150/thno.44523)
Supplement: Supplementary file 1 — Supplementary figures and tables. [file thnov10p7510s1.pdf]

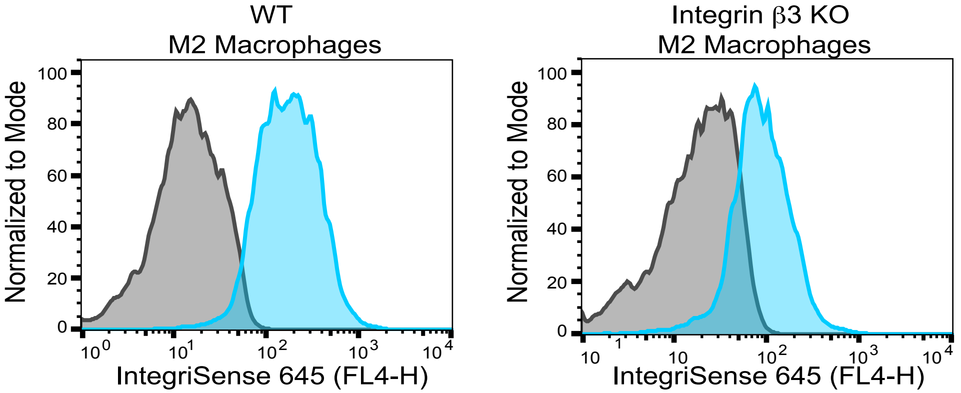


Figure S1. Targetable αvβ3 is expressed in greater amounts on M2 macrophages following incubation with an αvβ3 integrin specific fluorescent probe (Integrasense 645, PerkinElmer) *in vitro*.

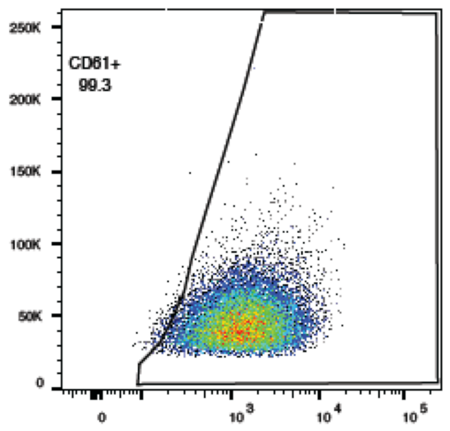


Figure S2. Flow cytometry analysis of β3 integrin expression in the 4T1.GFP.FL breast cancer cell line.

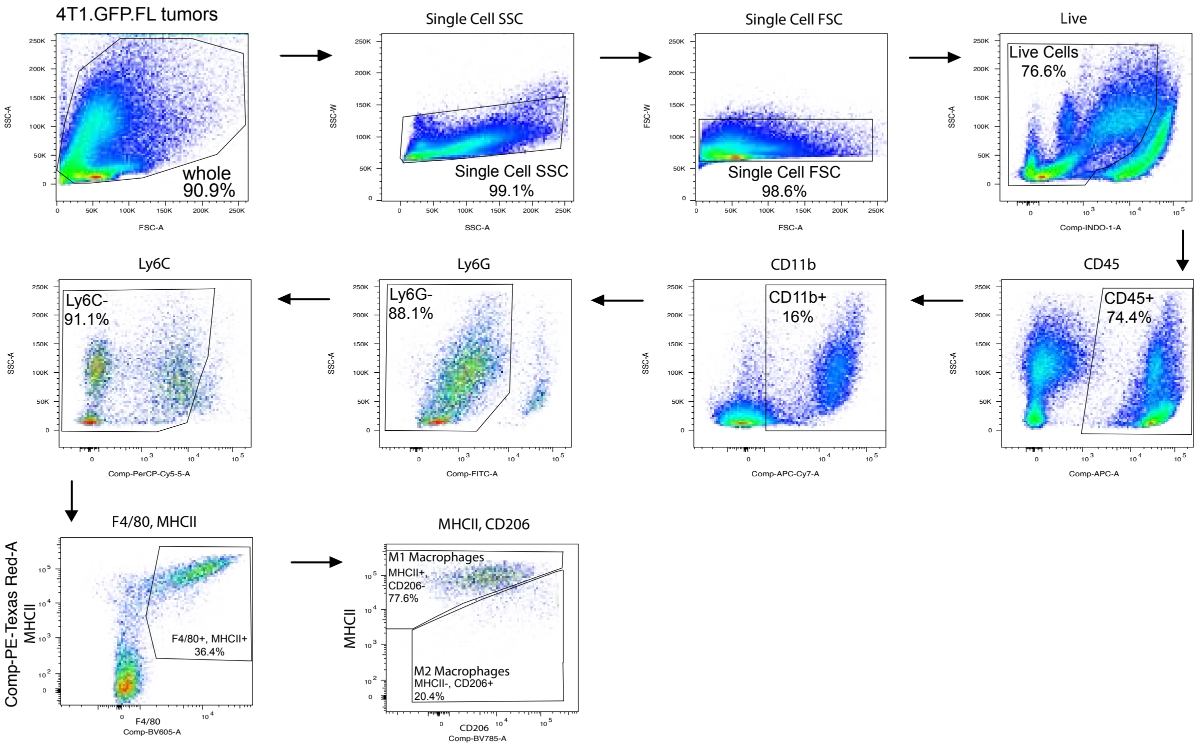


Figure S3. Flow cytometry analysis of immune popoulations isolated from mammary fat pad tumors estabished with the murine breast cancer cell line 4T1.GFP.FL.

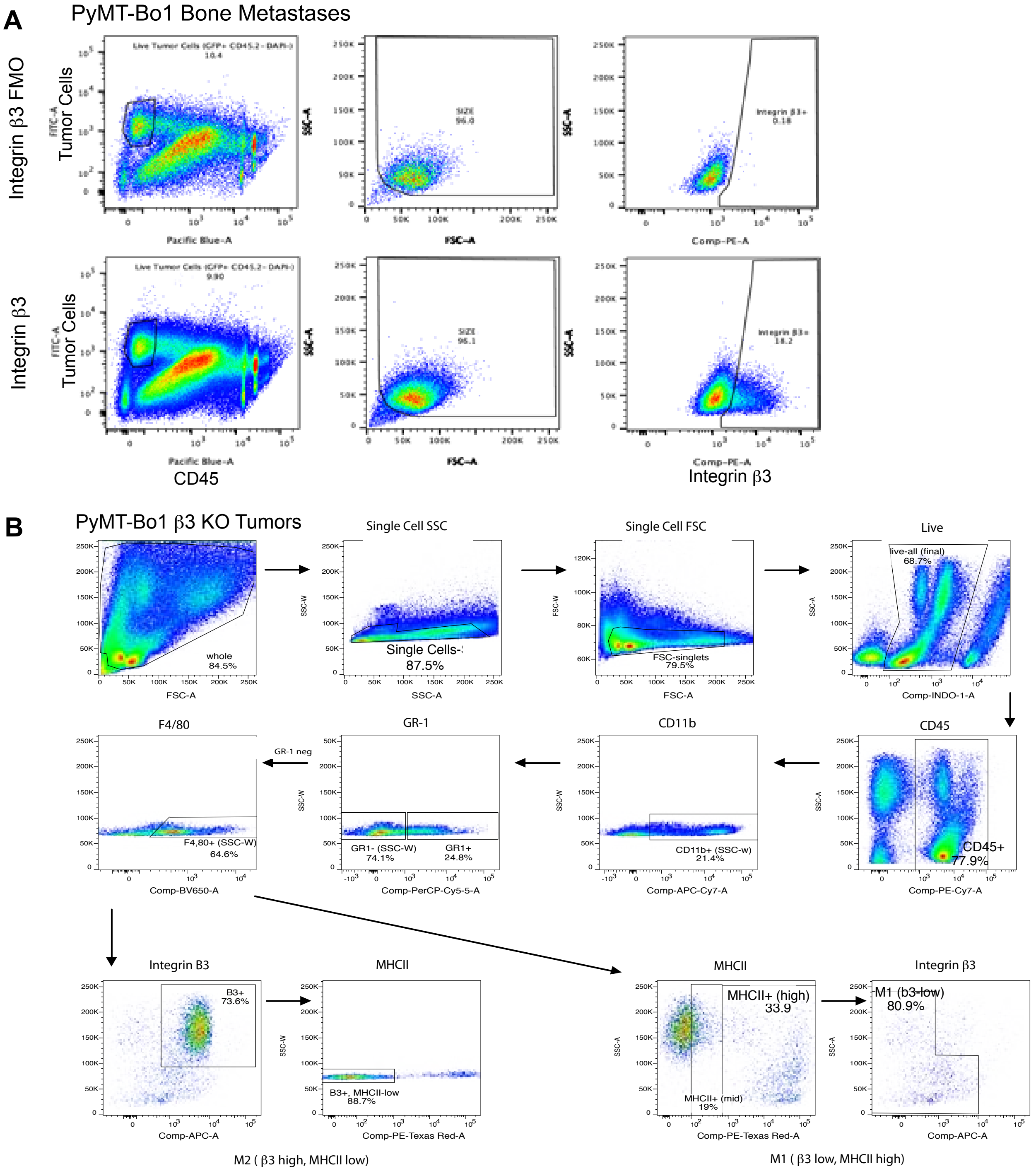


Figure S4. Flow cytometry gating strategies. (A) Flow cytometry analysis of integrin β3 expression of tumor cells isolated from the PyMT-Bo1 muring model of bone metastases. (B) Flow cytometry analysis of immune popoulations isolated from mammary fat pad tumors estabished with the murine breast cancer cell line PyMT-Bo1with integrin β3 knocked out.

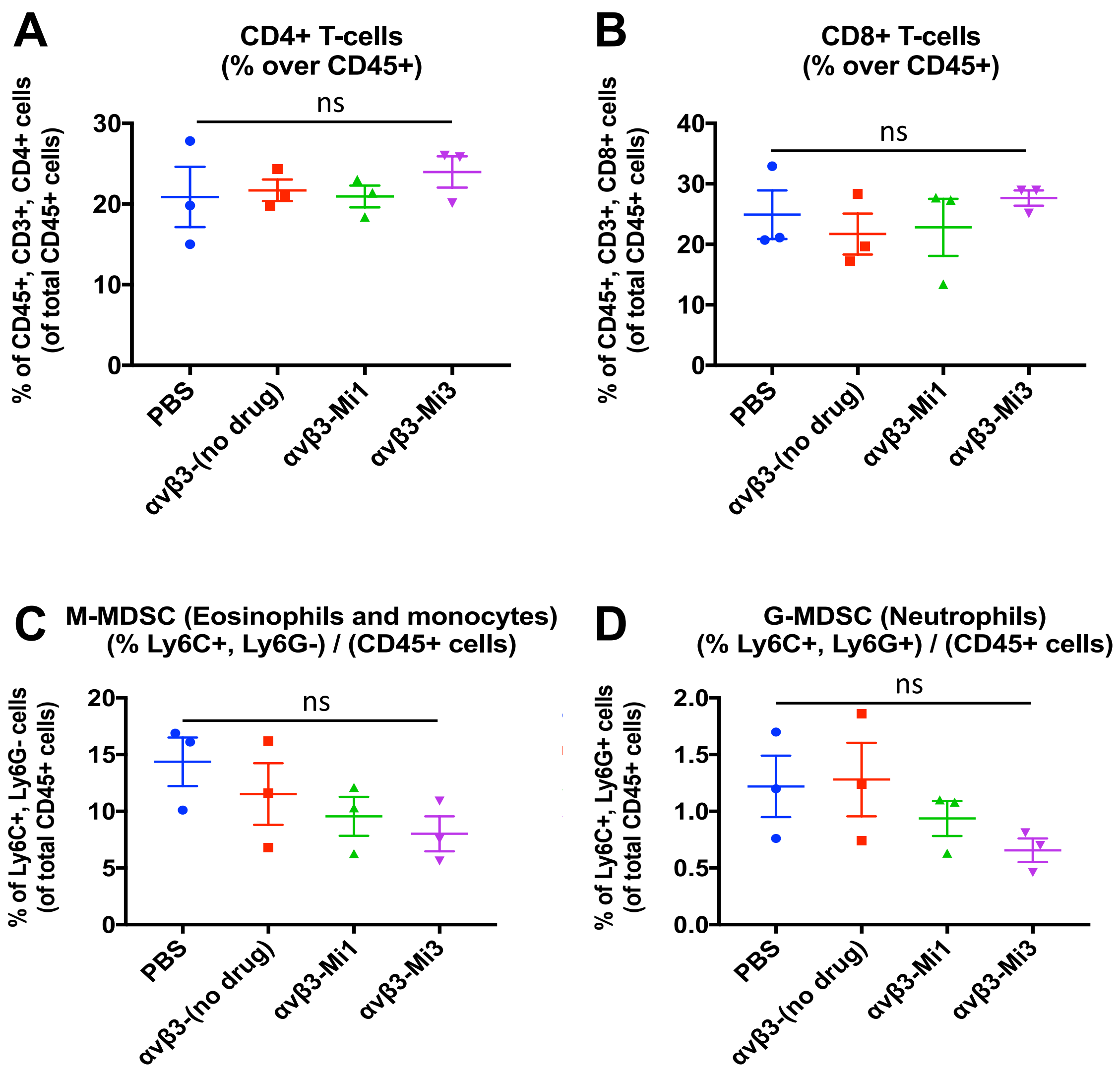


Figure S5. αvβ3-MI3-PD NP treated mammary fat pad tumors showed no significant difference in (A) CD4 T cells (B) CD8 T cells (C) M-MDSC or (D) G-MDSC immune cell populations.

| Description | Gene name | Entrez ID |
|---|---|---|
| c-Myc | MYC | ENSG00000136997 |
| Max | MAX | ENSG00000125952 |
| Cytokeratin 7 | KRT7 | ENSG00000135480 |
| Cd11b | CD11b | ENSG00000169896 |
| CSF1 receptor | CSF1R | ENSG00000182578 |
| CD47 | CD47 | ENSG00000196776 |

Table S1. RNA sequencing primers

| Gene | Forward Primer | Reverse Primer |
|---|---|---|
| c-Myc | CCCTAGTGCTGCATGAGGA | CCTCTTCTCCACAGACACCA |
| Max | ACCGAGGTTTCAATCTGCG | AGTCCCGCAAACTGTGAAAG |
| Actin | CTGTATTCCCCTCCATCGTG | CCTCGTCACCCACATAGGAG |
| Wnt5a | TCAGGACCACATGCAGTACAT | TGTCCACTGTGCTGCAGTTC |
| AKAP12 | TGCAATCTGCTTTGTCTTGG | GCCAGTGAAGAACATGAGCA |
| MAOA | GCCAGGAACGGAAATTTGTA | TCTCAGGTGGAAGCTCTGGT |
| MRC1 | GCAAATGGAGCCGTCTGTGC | CTCGTGGATCTCCGTGACAC |
| PCSK5 | AGTAGGTTGACTGGGACTGG | AGATCGCATAGCCAGCAAGT |

Table S2. Murine RT-PCR Primers

| Target | Fluorophore | Antibody |
|---|---|---|
| CD45 | PE-Cy7 | Clone: 104 |
| CD11b | APC-efluor780 | Clone: M1/70 |
| GR1 | PerCP-Cy5.5 | Clone: RB6-8C5 |
| F4/80 | BV650 | Clone: BM8 |
| MHCII | PE/Dazzle 594 | Clone: M5-114.15.2 |
| CD206 | BV421 | Clone: C068C2 |
| Integrin B3 | AF647 | Clone: 2C9.G2 |
| Tumor Cell | GFP | |
| Live/Dead Blue | 450/50 | |

Table S3. Antibody panel for flow cytometry analysis of cell populations derived from mammary fat pad tumors.

Supplemental Methods

The present manuscript focuses on the effectiveness of cMYC-MAX dimer antagonism to reduce the influence of tumor promoting M2 macrophages within the TAM population. This brief summary provides the reader access to some of the scientific literature regarding perfluorocarbon nanoparticles (PFC) and the drug delivery mechanism, we term contact-facilitated drug delivery. Note that the references for this section are cited below, separate for those of the paper proper.

The medical history of perfluorocarbon (PFC) and PFC emulsions has an extensive scientific literature dating back to 1966 [1]. PFCs are chemically at ground state and nonreactive [2]. They are neither hydrophilic or lipophilic [2]. PFC particle sizes are therefore very stable, dependent on the surfactants. They do not imbibe water and swell. Over time, the degradation of PFC particles occurs by a process known as Ostwald ripening. Ostwald ripening occurs when components of a discontinuous phase (PFC particles) diffuse through the continuous phase (aqueous media) from smaller to larger droplets. PFC emulsions have long shelf-lives at room temperature, which afforded the use in clinical studies. PFC nanoparticles have high biocompatibility because PFCs are unreactive and unmetabolized in the body [3]. This is due to the dense electron cloud surrounding the C-F bonds. PFC, PFOB (perfluorooctylbromide) in this manuscript, are eliminated, in man, as PFC gas through the lungs. While chemically inert, PFOB and other PFCs have the capacity to dissolve oxygen, which was dramatically demonstrated by liquid-ventilation, i.e., liquid breathing, in rodents in 1966 [1]. Continued research with PFC emulsions (nanoparticles, [NP]) [4-10] led to a seminal clinical demonstration of improved pulmonary function in neonatal infants suffering with respiratory distress syndrome [11]. These fragile neonates received intratracheal PFC NP and experienced improved respiratory function with negligible adverse effects [11]. However, during clinical testing, artificial

lung surfactant replacement technology emerged as a competitive concept and quickly displaced the intratracheal PFC approach.

Sterile PFOB emulsions were produced by Alliance Pharmaceuticals [3, 12, 13] and PFDCO (perfluorodichlorooctane) emulsions and developed by HemaGen/PFC [14]. Both formulations completed GLP safety and stability development and were produced by GMP manufacture for late stage clinical trials in the US and ex-US. These indications included neonates with respiratory distress and acute use as artificial blood substitutes. Unfortunately, the oxygen release profile of PFC nanoparticles did not mimic the hysteresis of hemoglobin dissociation curves exhibited by erythrocytes. Consequently, they failed to provide adequate oxygen delivery to ischemic tissues.

Over the last 25 years, PFC NPs have been extensively explored in both preclinical and clinical realms. Initial applications involved IV administration of the vascular-constrained PFC NP for blood pool imaging (MR, ultrasound, CT), reticuloendothelial (RES) organ imaging, gastrointestinal imaging [15-22], inflammation imaging [15-22] and cell tracking [23]. Lanza, Wickline, et al were the first to functionalize PFC nanoparticles for molecular imaging and later drug delivery (i.e., so-called theranostics) for many pathologies [24-34]. Kereos, Inc., co-founded by the Lanza/Wickline lab, translated two αvβ3-PFC pharmaceutical candidates to the clinic: one in Australia (Dr. Hodsman, Melbourne, VIC, PRO-KI02-06-0308-0162.00, 2008) and the other in the US (IND# 108320, Dr. Rich, Washington University Medical School). These GMP particles underwent extensive CMC characterization as well as formal GLP stability, toxicology and efficacy in multiple species. The PFC nanoparticles studied in the present manuscript are the same as those that developed for the clinic. However, in the present manuscript a very small amount (5 mol%) of Sn2 prodrug is included in the phospholipid membrane.

The spherical appearance of targeted PFC nanoparticles has been presented with scanning electron microscopy (SEM) bound to fibrin-rich thrombus [35] to upregulated tissue factor on proliferating vascular smooth muscle cells [36], and to various cell types showing the hemifusion mechanism of CFDD. A TEM photomicrograph of αvβ3-PFOB-MYC-PD NP, as used in this manuscript, was previously reported in 2015 by Pan et al. [37].

The concept of contact facilitated drug delivery (CFDD) has been a cornerstone of our drug delivery efforts since 2002 [38]. We have reviewed the concept in detail and published several papers demonstrating how the mechanism bypasses the endosomal pathway [39-43] by fusing with the outer leaflet of the target cell.  These studies were demonstrated with fluorescent phosphatidylethanolamine membrane biomarkers, fusogenic cytolytic peptides, and phosphatidylcholine Sn 2 cyanine dyes and doxorubicin prodrugs and have imaging data examples using fluorescent microscopy, confocal fluorescent microscopy, fluorescent life-time imaging, single molecule super high resolution microscopy and electron microscopy (SEM) are presented.

As mentioned previously, the systemic clearance of PFC nanoparticles is via the reticuloendothelial system with eventual elimination by exhalation in man. The accumulation of PFC in the spleen and livers of patients and animal models was known since systemic studies were performed [3, 14, 44]. At very high doses used for artificial blood, the particles elicited flu-like symptoms due to cytokine release from engorged phagocytes, which was sometimes ameliorated with adjunctive steroids or nonsteroidal anti-inflammatory medications in patients. Phagocytosis by macrophages leads to enzymatic metabolism of surfactant components. However, the subset of particles that home to the αvβ3-integrin presented by the M2-like macrophages undergo rapid irreversible membrane fusion and direct translocation of the prodrug into the cells, by passing the phagocytic pathway.

The development of the Sn 2 prodrugs in the context of CFDD in our lab occurred when pharmacokinetic studies tracking fumagillin and other drugs relative to the PFC core and the

αvβ3-homing ligand in circulation using HLPC-MS/MS showed inadequate retention of the dissolved drugs [45]. After extensive testing of different prodrug concepts, a Sn 2 approach was adopted to embed and protect the drugs in the lipid membrane during circulation and to release the drug into the cytosol after targeting and membrane hemifusion.

Prior research on Sn2 lipids was first conceived by David Thompson and further developed by Thomas Andressen et al in the context of liposomes as reviewed in Pan et al [46]. The use of this prodrug approach failed with liposomes, which required polyethylene glycol coatings to extend circulatory half-life. It was effective for nonpegylated PFC nanoparticles for two reasons. The first was that natural phospholipid membrane are resistant to water, including liposomes, unless they are pegylated. Pegylation creates a water corona around the particle that wicks into the membrane, allowing enzymes access to the glycerol ester bonds. This initiates rapid premature drug release. The second, specifically for PFC particles, is the fact, demonstrated by computational simulation of the particle self-assembly process, which indicated that the perfluorocarbon core penetrates between the phospholipids to the particle-water interface [47]. Water is immiscible with PFC; almost nothing is miscible in PFC. We have previously demonstrated that the Sn2-lipase labile prodrugs (docetaxel) are stable in PFOB particles using dissolution with HPLC [48]. Only after the PFC nanoparticle is "cracked" with isopropyl alcohol in the presence of plasma or excess phospholipase enzyme can the prodrug be metabolized.

1. Clark LC, Jr., Gollan F. Survival of mammals breathing organic liquids equilibrated with oxygen at atmospheric pressure. Science. 1966; 152: 1755-6.

2. Dixon DD, Holland DG. Fluorocarbons: properties and syntheses. Fed Proc. 1975; 34: 1444-8.

3. Flaim S. Pharmacokinetics and side effects of perfluorocarbon-based blood substitutes. Art Cells Blood Subs and Immob Biotech. 1994; 22: 1043-54.

4. Fuhrman BP, Paczan PR, DeFrancisis M. Perfluorocarbon-associated gas exchange. Crit Care Med. 1991; 19: 712-22.

5. Tutuncu AS, Faithfull NS, Lachmann B. Intratracheal perfluorocarbon administration combined with mechanical ventilation in experimental respiratory distress syndrome: dose-dependent improvement of gas exchange. Crit Care Med. 1993; 21: 962-9.

6. Hirschl RB, Overbeck MC, Parent A, Hernandez R, Schwartz S, Dosanjh A, et al. Liquid ventilation provides uniform distribution of perfluorocarbon in the setting of respiratory failure. Surgery. 1994; 116: 159-67; discussion 67-8.

7. Wilcox DT, Glick PL, Karamanoukian HL, Leach C, Morin FC, 3rd, Fuhrman BP. Perfluorocarbon-associated gas exchange improves pulmonary mechanics, oxygenation, ventilation, and allows nitric oxide delivery in the hypoplastic lung congenital diaphragmatic hernia lamb model. Crit Care Med. 1995; 23: 1858-63.

8. Papo MC, Paczan PR, Fuhrman BP, Steinhorn DM, Hernan LJ, Leach CL, et al. Perfluorocarbon-associated gas exchange improves oxygenation, lung mechanics, and survival in a model of adult respiratory distress syndrome. Crit Care Med. 1996; 24: 466-74.

9. Tutuncu AS, Houmes RJ, Bos JA, Wollmer P, Lachmann B. Evaluation of lung function after intratracheal perfluorocarbon administration in healthy animals. Crit Care Med. 1996; 24: 274-9.

10. Fitzpatrick JC, Jordan BS, Salman N, Williams J, Cioffi WG, Jr., Pruitt BA, Jr. The use of perfluorocarbon-associated gas exchange to improve ventilation and decrease mortality after inhalation injury in a neonatal swine model. J Pediatr Surg. 1997; 32: 192-6.

11. Salon JE. Perflubron in infants with severe respiratory distress syndrome. N Engl J Med. 1997; 336: 660.

12. Long DC, Long DM, Riess J, Follana R, Burgan A, Mattrey RF. Preparation and application of highly concentrated perfluoroctylbromide fluorocarbon emulsions. Biomater Artif Cells Artif Organs. 1988; 16: 441-2.

13. Flaim SF, Hazard DR, Hogan J, Peters RM. Characterization and mechanism of side-effects of Oxygent HT (highly concentrated fluorocarbon emulsion) in swine. Biomater Artif Cells Immobilization Biotechnol. 1991; 19: 383.

14. Kaufman R. Clinical development of perfluorocarbon-based emulsions as red cell substitutes. In: J S, editor. Emulsions and emulsion stability. New York: Marcel Dekker; 1996. p. 343-67.

15. Mattrey R, Scheible F, Gosink B, Leopold G, Long D, Higgins C. Perfluoroctylbromide: A liver/spleen=specific and tumor-imaging ultrasound contrast material. Radiology. 1982; 145: 759-62.

16. Mattrey RF, Long DM, Multer F, Mitten R, Higgins CB. Perfluoroctylbromide: a reticuloendothelial-specific and tumor-imaging agent for computed tomography. Radiology. 1982; 145: 755-8.

17. Mattrey RF, Leopold GR, van Sonnenberg E, Gosink BB, Scheible FW, Long DM. Perfluorochemicals as liver and spleen seeking ultrasound contrast agents. J Ultrasound Med. 1983; 2: 173-6.

18. Hackney D, Slutsky RA, Mattrey R, Peck WW, Abraham JL, Shabetai R, et al. Experimental pericardial inflammation evaluated by computed tomography. Radiology. 1984; 151: 145-8.

19. Mattrey RF, Andre M, Campbell J, Mitten R, Multer F, Hackney D, et al. Specific enhancement of intra-abdominal abscesses with perfluoroctylbromide for CT imaging. Invest Radiol. 1984; 19: 438-46.

20. Mattrey RF, Andre MP. Ultrasonic Enhancement of Myocardial Infarction with Perfluorocarbon Compounds in Dogs. Am J Cardiol. 1984; 54: 206-10.

21. Mattrey RF, Long DM, Peck WW, Slutsky RA, Higgins CB. Perfluoroctylbromide as a blood pool contrast agent for liver, spleen, and vascular imaging in computed tomography. J Comput Assist Tomogr. 1984; 8: 739-44.

22. Kono Y, Mattrey RF. Ultrasound of the liver. Radiol Clin North Am. 2005; 43: 815-26, vii.

23. Ahrens ET, Bulte JWM. Tracking immune cells in vivo using magnetic resonance imaging. Nat Rev Immunol. 2013; 13: 755-63.
24. Lanza G, Wallace K, Scott M, Cacheris W, Abendschein D, Christy D, et al. A novel site-targeted ultrasonic contrast agent with broad biomedical application. Circulation. 1996; 94: 3334-40.
25. Lanza GM, Wallace KD, Fischer SE, Christy DH, Scott MJ, Trousil RL, et al. High-frequency ultrasonic detection of thrombi with a targeted contrast system. Ultrasound Med Biol. 1997; 23: 863-70.
26. Lanza G, Lorenz C, Fischer S, Scott M, Cacheris W, Kaufman R, et al. Enhanced detection of thrombi with a novel fibrin-targeted magnetic resonance imaging agent. Acad Radiol. 1998; 5(suppl 1): s173-s6.
27. Yu X, Song SK, Chen J, Scott MJ, Fuhrhop RJ, Hall CS, et al. High-resolution MRI characterization of human thrombus using a novel fibrin-targeted paramagnetic nanoparticle contrast agent. Magn Reson Med. 2000; 44: 867-72.
28. Anderson SA, Rader RK, Westlin WF, Null C, Jackson D, Lanza GM, et al. Magnetic resonance contrast enhancement of neovasculature with alpha(v)beta(3)-targeted nanoparticles. Magn Reson Med. 2000; 44: 433-9.
29. Winter PM, Caruthers SD, Kassner A, Harris TD, Chinen LK, Allen JS, et al. Molecular imaging of angiogenesis in nascent Vx-2 rabbit tumors using a novel alpha(nu)beta3-targeted nanoparticle and 1.5 tesla magnetic resonance imaging. Cancer Res. 2003; 63: 5838-43.
30. Winter PM, Morawski AM, Caruthers SD, Fuhrhop RW, Zhang H, Williams TA, et al. Molecular imaging of angiogenesis in early-stage atherosclerosis with alpha(v)beta3-integrin-targeted nanoparticles. Circulation. 2003; 108: 2270-4.
31. Schmieder A, Winter P, Caruthers S, Harris T, Williams T, Allen J, et al. MR molecular imaging of melanoma angiogenesis with avb3-Targeted paramagnetic nanoparticles. Magn Reson Med. 2005; 53: 621-7.

32. Winter P, Neubauer A, Caruthers S, Harris T, Robertson J, Williams T, et al. Endothelial alpha(nu)beta(3)-Integrin targeted fumagillin nanoparticles inhibit angiogenesis in atherosclerosis. Arterioscler Thromb Vasc Biol 2006; 26: 2103 - 9.
33. Hu G, Lijowski M, Zhang H, Partlow KC, Caruthers SD, Kiefer G, et al. Imaging of Vx-2 rabbit tumors with alpha(nu)beta3-integrin-targeted 111In nanoparticles. Int J Cancer. 2007; 120: 1951-7.
34. Cyrus T, Zhang H, Allen JS, Williams TA, Hu G, Caruthers SD, et al. Intramural delivery of rapamycin with alphavbeta3-targeted paramagnetic nanoparticles inhibits stenosis after balloon injury. Arterioscler Thromb Vasc Biol. 2008; 28: 820-6.
35. Flacke S, Fischer S, Scott M, Fuhrhop R, Allen J, Mc Lean M, et al. A novel MRI contrast agent for molecular imaging of fibrin:implications for detecting vulnerable plaques. Circulation. 2001; 104: 1280 -5.
36. Lanza GM, Abendschein DR, Hall CS, Marsh JN, Scott MJ, Scherrer DE, et al. Molecular imaging of stretch-induced tissue factor expression in carotid arteries with intravascular ultrasound. Invest Radiol. 2000; 35: 227-34.
37. Pan D, Kim B, Hu G, Gupta DS, Senpan A, Yang X, et al. A strategy for combating melanoma with oncogenic c-Myc inhibitors and targeted nanotherapy. Nanomedicine (Lond). 2015; 10: 241-51.
38. Lanza GM, Yu X, Winter PM, Abendschein DR, Karukstis KK, Scott MJ, et al. Targeted antiproliferative drug delivery to vascular smooth muscle cells with a magnetic resonance imaging nanoparticle contrast agent: implications for rational therapy of restenosis. Circulation. 2002; 106: 2842-7.
39. Gregory M. Lanza, Xin Yu, Patrick M. Winter, Dana R. Abendschein, Kerry K. Karukstis, Michael J. Scott, et al. Targeted antiproliferative drug delivery to vascular smooth muscle cells with a magnetic resonance imaging nanoparticle contrast agent: implications for rational therapy of restenosis. Circulation. 2002; 106: 2842 - 7.

40. Partlow K, Lanza G, Wickline S. Exploiting lipid raft transport with membrane targeted nanoparticles: A strategy for cytosolic drug delivery. Biomaterials 2008; 29: 3367-75.
41. Soman N, Lanza G, Heuser J, Schlesinger P, Wickline S. Synthesis and characterization of stable fluorocarbon nanostructures as drug delivery vehicles for cytolytic peptides. Nano Lett. 2008; 8: 1131-6.
42. Soman N, Marsh J, Lanza G, Wickline S. New mechanisms for nonporative ultrasound stimulation of cargo delivery to cell cytosol with targeted perfluorocarbon nanoparticles. Nanotechnology. 2008; 19 185102-7.
43. Maji D, Lu J, Sarder P, Schmieder A, Cui G, Yang X, et al. Cellular trafficking of Sn-2 phosphatidylcholine prodrugs studied with fuorescence lifetime imaging and super-resolution microscopy. Prec Nanomed. 2018; 1: 128-45.
44. Flaim SF, Hazard DR, Hogan J, Peters RM. Characterization and mechanism of side-effects of Oxygent HT (highly concentrated fluorocarbon emulsion) in swine. Biomater Artif Cells Immobilization Biotechnol. 1994; 22: 1511-5.
45. Pan D, Sanyal N, Schmieder AH, Senpan A, Kim B, Yang X, et al. Antiangiogenic nanotherapy with lipase-labile Sn-2 fumagillin prodrug. Nanomedicine (Lond). 2012; 7: 1507-19.
46. Pan D, Pham CT, Weilbaecher KN, Tomasson MH, Wickline SA, Lanza GM. Contact-facilitated drug delivery with Sn2 lipase labile prodrugs optimize targeted lipid nanoparticle drug delivery. Wiley Interdiscip Rev Nanomed Nanobiotechnol. 2016; 8: 85-106.
47. Lee SJ, Olsen B, Schlesinger PH, Baker NA. Characterization of perfluorooctylbromide-based nanoemulsion particles using atomistic molecular dynamics simulations. J Phys Chem B. 2010; 114: 10086-96.
48. Pan D, Schmieder AH, Wang K, Yang X, Senpan A, Cui G, et al. Anti-angiogenesis therapy in the Vx2 rabbit cancer model with a lipase-cleavable Sn 2 taxane phospholipid prodrug using alpha(v)beta(3)-targeted theranostic nanoparticles. Theranostics. 2014; 4: 565-78.
